# Estradiol resolves pneumonia via ER**β** in regulatory T cells

**DOI:** 10.1172/jci.insight.133251

**Published:** 2021-02-08

**Authors:** Ye Xiong, Qiong Zhong, Tsvi Palmer, Alison Benner, Lan Wang, Karthik Suresh, Rachel Damico, Franco R. D’Alessio

**Affiliations:** Division of Pulmonary Critical Care Medicine, Johns Hopkins University School of Medicine, Baltimore, Maryland, USA.

**Keywords:** Immunology, Pulmonology, Bacterial infections, Cellular immune response, T cells

## Abstract

Current treatments for pneumonia (PNA) are focused on the pathogens. Mortality from PNA-induced acute lung injury (PNA-ALI) remains high, underscoring the need for additional therapeutic targets. Clinical and experimental evidence exists for potential sex differences in PNA survival, with males having higher mortality. In a model of severe pneumococcal PNA, when compared with male mice, age-matched female mice exhibited enhanced resolution characterized by decreased alveolar and lung inflammation and increased numbers of Tregs. Recognizing the critical role of Tregs in lung injury resolution, we evaluated whether improved outcomes in female mice were due to estradiol (E2) effects on Treg biology. E2 promoted a Treg-suppressive phenotype in vitro and resolution of PNA in vivo. Systemic rescue administration of E2 promoted resolution of PNA in male mice independent of lung bacterial clearance. E2 augmented Treg expression of Foxp3, CD25, and GATA3, an effect that required ERβ, and not ERα, signaling. Importantly, the in vivo therapeutic effects of E2 were lost in Treg-depleted mice (*Foxp3^DTR^* mice). Adoptive transfer of ex vivo E2-treated Tregs rescued *Streptococcus* *pneumoniae*–induce PNA-ALI, a salutary effect that required Treg ERβ expression. E2/ERβ was required for Tregs to control macrophage proinflammatory responses. Our findings support the therapeutic role for E2 in promoting resolution of lung inflammation after PNA via ERβ Tregs.

## Introduction

Pneumonia (PNA) is one of the leading causes of death worldwide, resulting in an estimated 2.74 million deaths (1). PNA can result in devastating acute inflammatory injury in the lung, manifesting in acute lung injury (ALI), clinically known as acute respiratory distress syndrome (ARDS). PNA can also worsen underlying comorbid conditions, and it is associated with increased mortality months to years after the initial inciting event (2). Current treatments for ALI/ARDS caused by PNA are focused primarily on pathogen killing with antibiotics but do not target excessive lung inflammation elicited by the host immune response (3). Given the morbidity and mortality of PNA, even in the era of broadly available antibiosis and vaccinations, there is an urgent need for additional biological insight into the host responses to inflammation caused by incident PNA. Unfortunately, in part due to lack of mechanistic clarity, we currently have little understanding of how to modulate host factors in order to improve PNA outcomes.

Clinical and experimental evidence exists for sex differences in PNA outcomes, with males having higher severity (4) and mortality (4–6). Both humoral and cellular immune responses are known to be more active in females than in males (7, 8). The burden of infectious diseases is generally higher in men than in women (9–11). As a result, responses to vaccines and clearance of pathogens are enhanced in females compared with males, although this could lead to exuberant inflammatory responses in the female host (8, 12). Sex differences in immune responses are complex and can be explained by differential chromosomal organization, sex steroid levels, hormonal receptor distribution and expression in different cells, relative difference in immune cell composition, and differential expression of pattern recognition receptor environmental exposures, which can influence microbiome composition (8).

In these studies, we focused on estradiol (E2), which plays a significant role in regulating immunologic sex differences (13, 14). E2 has been implicated in the regulation of numerous inflammatory disorders (15), such as autoimmune encephalomyelitis (16) and severe acute respiratory syndrome coronavirus infection (17). E2 is protective in experimental systemic LPS and intestinal ischemic-induced ALI (18–20) when administered before the onset of the inflammatory insult. For experimental PNA, the role of E2 remains disparate. Protective effects have been described in a mouse pneumococcal PNA model (21, 22), whereas a proinflammatory role was observed in a pseudomonas aeruginosa PNA model (23). Studies of the protective effects of E2 have mostly focused on early phases of lung injury; to our knowledge, a potential role for estrogen in the resolution of PNA not been investigated.

Tregs comprise a small yet potent subpopulation of CD4^+^ lymphocytes (5%–10%) in humans and mice (24, 25). Tregs maintain immunological self-tolerance and homeostasis by suppressing aberrant or excessive immune responses (26, 27). They express high levels of CD25 (the IL-2Rα subunit) and the forkhead box protein 3 (Foxp3) master transcription factor (28). We previously showed that CD4^+^CD25^+^Foxp3^+^ Tregs resolve experimental ALI by modulating the following critical prorepair steps: (a) abrogation of macrophage proinflammatory responses (29), (b) augmentation of neutrophil efferocytosis (29), (c) limitation of fibroproliferation (30), and (d) augmentation of alveolar epithelial repair (31). In summary, Tregs are pivotal prorepair cells after ALI (32, 33). Moreover, in a previous study, we identified Tregs in the alveolar spaces of patients with ALI (29, 34), and a recent study found increased Treg numbers in survivors of ALI compared with those who did not survive (35). Exogenous E2 has been shown to expand Tregs in mice (36). Treg pro-repair function could lead to development of Treg-based therapeutics for injured lungs (37, 38).

To investigate the role of E2 in the resolution phase of PNA, we established a model of resolution following pneumococcal-induced lung injury using intratracheal *Streptococcus* *pneumoniae* (PNA-induced ALI [PNA-ALI]). We found that male and female mice have comparable early lung inflammatory responses to *S*. *pneumoniae*. Despite similar bacterial burdens in the lung, male mice had sustained lung injury 6 days after initial infection and prolonged elevation of observed bronchoalveolar lavage (BAL) inflammatory expression of cytokines, including IFN-γ, TNF-α, IL-6 and IL-12p70. Further, Treg numbers in both the BAL and lung were increased in female mice in the resolution phase of PNA. Exogenous systemic administration of E2 given as rescue treatment 48 hours after lung infection promoted resolution of PNA-ALI in male mice with decreased lung inflammation, decreased BAL inflammatory cytokines, and increased the number of lung Tregs. This was also independent of effects on lung bacterial burden. The salutary effects of exogenous E2 were lost in Treg-depleted animals. Isolated Tregs stimulated in vitro with E2 showed an enhanced suppressive phenotype characterized by upregulation of their master transcription factor Foxp3, GATA3, surface expression of IL-2Rα (CD25), and glucocorticoid-induced TNF receptor (GITR) expression. CD4^+^CD25^–^ T conventional cells did not upregulate any of these markers in response to E2. *ERα^–/–^* but not *ERβ^–/–^* Tregs responded similarly to E2 as WT Tregs, suggesting *ERβ* requirements in Treg estrogenic stimulation. To determine the functional necessity for the *ERβ* receptor on Tregs, lymphocyte-deficient mice were treated with subtherapeutic doses of Tregs after *S*. *pneumoniae* injury. Animals were randomized to E2-stimulated Tregs derived from WT or *ERβ^–/–^* mice. Beneficial effects of E2-treated Tregs were dependent upon *ERβ^–/–^* expression. In vitro coculture studies demonstrate that the ability of Tregs to modify macrophage antiinflammatory IL-10 production was augmented in E2-treated Tregs and the Treg ERβ contributes to suppression of macrophage-generated proinflammatory cytokines. Our results provide support of estrogenic mechanisms involved in PNA-ALI resolution, identifying new targets regulating Treg function and therapeutics that enhance Treg prorepair function.

## Results

### Female sex displayed enhanced resolution of PNA.

**Figure 1:**
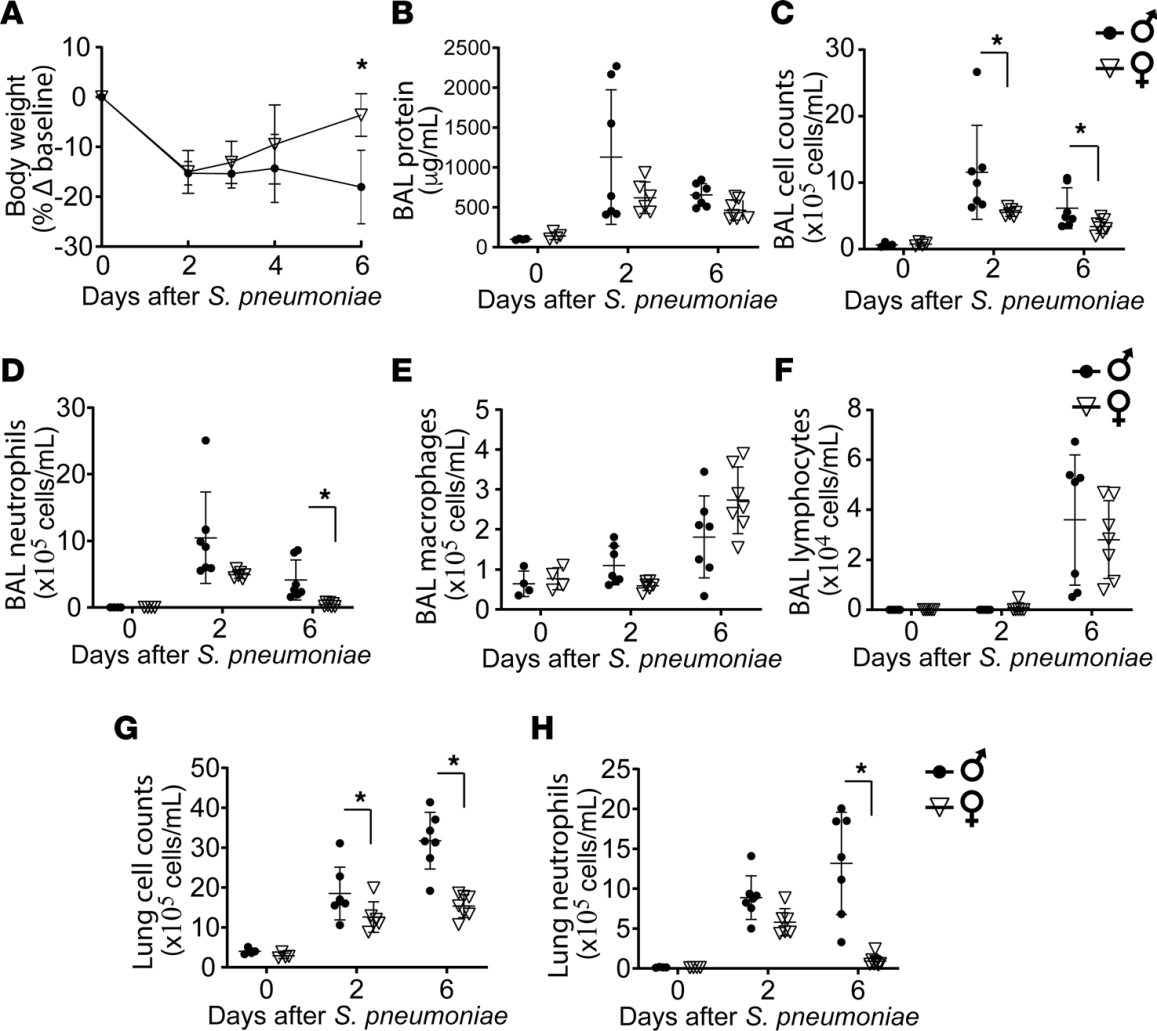
Female mice display enhanced resolution of pneumonia. Age-matched WT male and female mice were challenged with intratracheal *S*. *pneumoniae* (4 × 10^6^ CFU/mouse) and followed over time. Lung injury parameters were assessed on days 0, 2, and 6. (**A**) Body weight over time relative to baseline at day 0. (**B**–**D**) BAL total protein (**B**), BAL total cell count (**C**), and BAL differential cell counts (**D**–**F**) were determined over time in female and male WT mice after intratracheal *S*. *pneumoniae*. (**G** and **H**) Total lung total cell counts and lung neutrophil counts were determined in female and male mice after intratracheal *S*. *pneumoniae*. Two-way ANOVA was used. *n* = 6–7 per group per time point. *P < 0.05. Values are reported as mean ± SEM.

Given prior reports showing increased Foxp3 expression and suppressive function effect by E2 on Tregs (36), a cell type we previously showed to be important for ALI resolution (29), we hypothesized that resolution of PNA would be enhanced in female mice in vivo. Age-matched WT male and female mice were challenged with *S*. *pneumoniae*. Although male mice displayed higher total BAL and lung cell counts 2 days after PNA, their early lung inflammatory response was similar to that of female mice, as shown by similar increases in BAL protein and BAL neutrophil counts (Figure 1, B and C). While lung inflammation was largely cleared by 6 days after PNA-ALI in female mice, male mice experienced sustained weight loss (Figure 1A) and a higher number of BAL total cells (Figure 1C), with predominance of neutrophils (Figure 1D). Moreover, the proportion of alveolar cells showed a higher percentage of neutrophils and a lower percentage of macrophages in the male host by day 6 after PNA (Supplemental Figure 1; supplemental material available online with this article; https://doi.org/10.1172/jci.insight.133251DS1). The lung compartment profile of sustained inflammatory cells was similar to the alveolar compartment (Figure 1, G and H). The number of alveolar macrophages and lymphocytes was similar at all intervals after injury in male mice compared with female mice (Figure 1, E and F). Analysis of sex differences in BAL cytokines at over time during PNA demonstrated similar BAL proinflammatory cytokine profiles in both sexes at day 2 of PNA. In contrast, by day 6, female mice had significantly lower BAL IFN-γ, IL-12, IL-6, and TNF-α when compared with male mice (Supplemental Figure 2). These data suggest that female mice, in contrast to male mice, displayed enhanced resolution of similar lung inflammation after *S*. *pneumoniae*.

### Alveolar and lung Tregs increased in female mice with resolving PNA.

**Figure 2:**
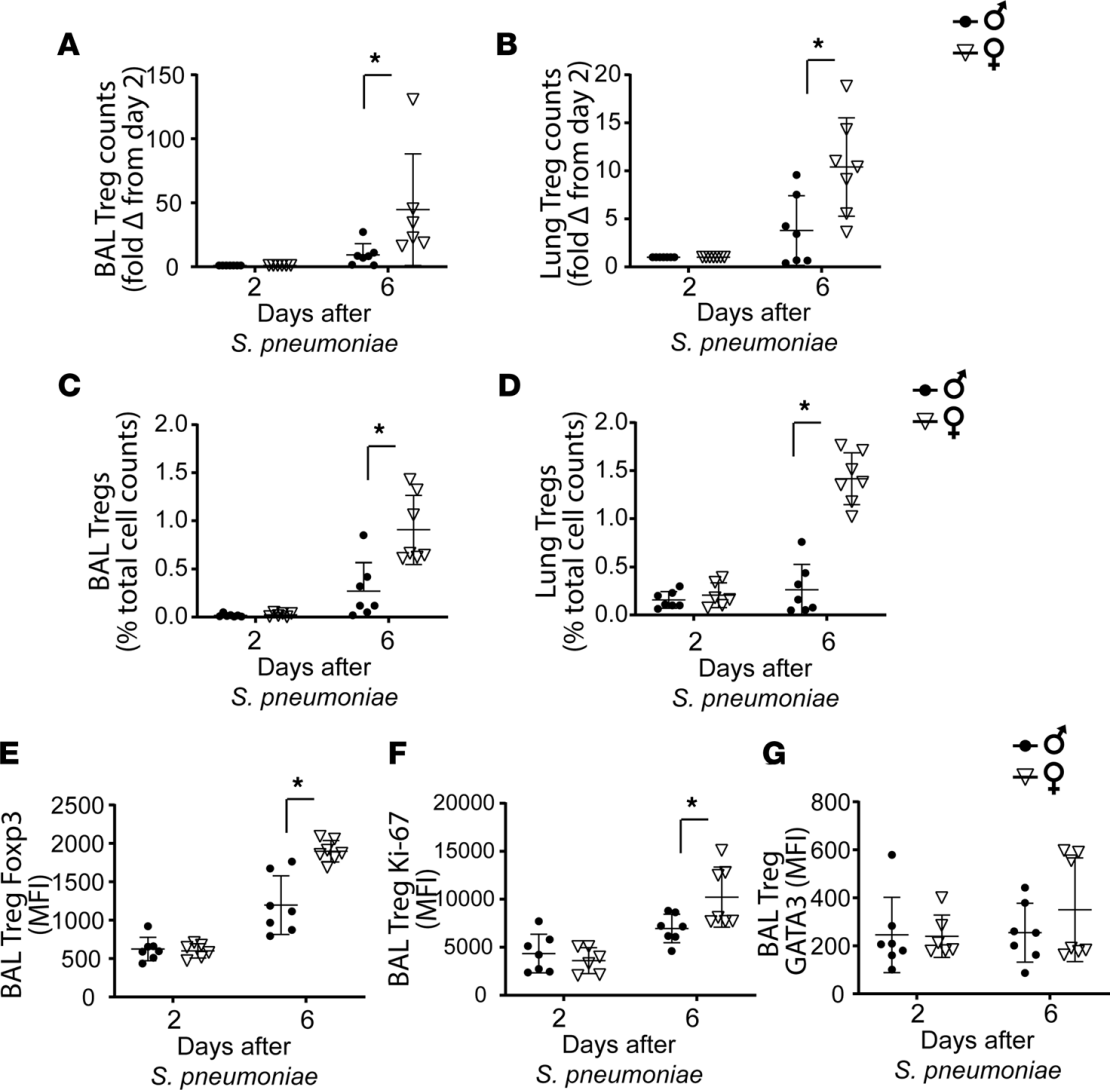
Alveolar and lung Tregs increased in female mice with resolving PNA. BAL and lung Treg numbers, suppressive phenotype, and proliferative capacity were measured by flow cytometry in male and female WT animals on days 2 and 6 after intratracheal *S*. *pneumoniae–*induced lung injury. (**A**–**D**) Fold change in female animals for BAL Treg (**A**) and lung Treg (**B**) numbers as well as BAL (**C**) and lung (**D**) Treg percentage compared with male levels at day 2 after *S*. *pneumoniae*. BAL Treg expression of master transcription factor Foxp3 (**E**), proliferative state by intracellular Ki-67 (**F**), and transcription factor GATA3 expression (**G**) were determined by mean fluorescence intensity and compared over time. Normalization followed by 2-way ANOVA. *n* = 6–7 per group per time point. *P < 0.05. Values are reported as mean ± SEM.

We next examined baseline Treg numbers and function in male and female mice in alveolar and lung compartments. At baseline, no differences in BAL and lung cell counts were observed. Lung Treg numbers were similar in both sexes at baseline (Supplemental Figure 3). Baseline expression of Treg glucocorticoid-induced TNFR-related protein (GITR) in the lungs of female mice was higher than that in male mice, while both sexes had similar Treg expression for Foxp3, CD25, and GATA3 (Supplemental Figure 3). During resolution, *S*. *pneumoniae*–injured female mice displayed a higher absolute fold increase in the number of alveolar (Figure 2A) and lung Tregs (Figure 2B) compared with their male counterparts. The proportion of Tregs in alveolar and lung compartments was significantly increased compared with that in male mice at day 6 (Figure 2, C and D). Notably, Foxp3 (Figure 2E) and Ki-67 (Figure 2F) expression was higher in female mice than in male mice during resolution, indicating an enhanced proliferative state. Treg GATA3 expression was similar in male and female mice (Figure 2G). Consistent with enhanced lung injury resolution, female mice demonstrated increased Treg numbers in both the lung and alveolar compartment. Further, markers of suppressive phenotype were enhanced in female Tregs relative to male Tregs.

### Exogenous estrogen enhanced the suppressive phenotype of Tregs.

**Figure 3:**
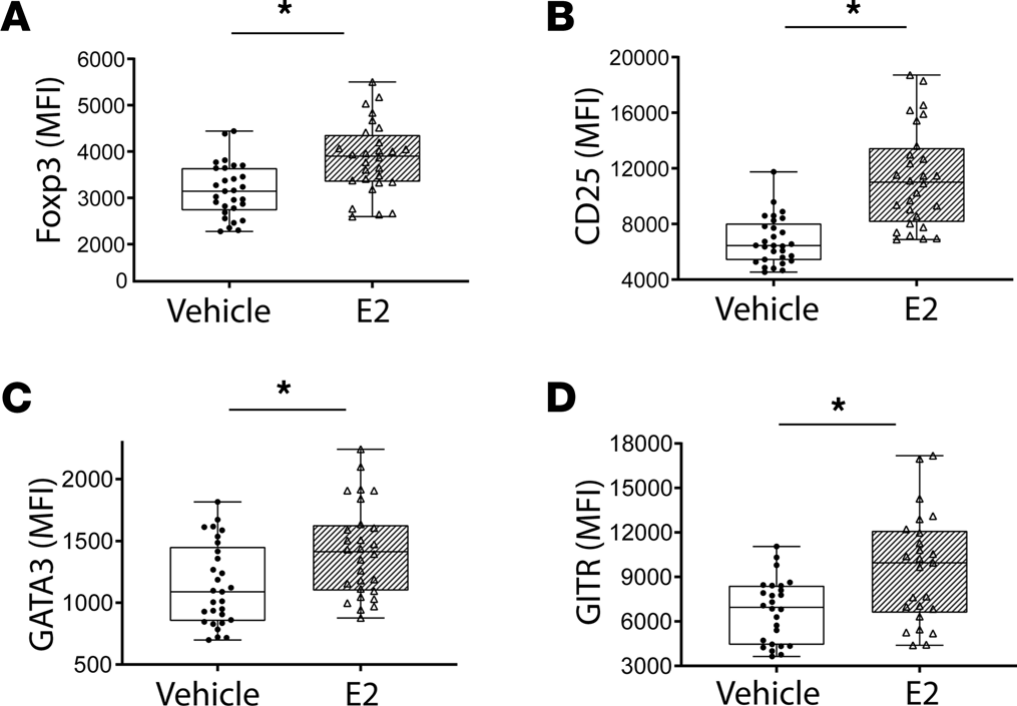
Estrogen enhances the Treg-suppressive phenotype in vitro. CD4^+^CD25^+^ Tregs were isolated from WT mouse splenocytes and cultured in the presence of anti-CD3/CD28 beads and stimulated with either vehicle or estradiol (E2; 10 μM) for 72 hours. Multicolor flow cytometry was performed to asses E2-dependent changes in Treg-suppressive phenotype. Treg expression for Foxp3 (**A**), CD25 (**B**), GATA3 (**C**), and GITR (**D**) was measured and is expressed as mean fluorescence intensity (MFI) ± SEM. The Mann-Whitney test was used for all MFI. *n* = 25–30 per group. *P < 0.01.

To determine the effect of exogenous E2 on Tregs, we cultured the CD4^+^CD25^+^ (Tregs, >85% Foxp3^+^) cells with anti-CD3/CD28 beads and stimulated them with either E2 or vehicle for 72 hours, prior to measuring markers associated with the Treg-suppressive phenotype. E2 treatment increased expression of Treg master transcription factor, Foxp3 (Figure 3A). Similarly, E2 increased CD25 (IL-2Rα) expression in Tregs (Figure 3B). Moreover, expression of proteins GATA3 and GITR was increased in E2-stimulated Tregs (Figure 3, C and D). GATA3 is a transcription factor known for its role on the migration of Tregs to inflamed sites, while GITR enhances proliferation of functionally competent Tregs. Other Treg markers known to play important roles in Treg biology but not altered by E2 stimulation include Ki-67, CD62L, CD69, CD39, PD-1, CTLA-4, CD44, and CD40L (Supplemental Figure 4). In order to determine if the effects of E2 are specific for Tregs, we evaluated the effect of E2 treatment on cultured conventional CD4^+^ T cells (CD4^+^CD25^–^; <1% Foxp3^+^). In contrast to Tregs, E2 had no effects on Foxp3, GATA3 (Supplemental Figure 5), CD25, or GITR expression (data not shown). It is worth noting that the effect of E2 on Tregs was independent of the presence of exogenous IL-2 (data not shown). These results showed that exogenous E2 robustly enhanced the Treg-suppressive phenotype in vitro.

### Therapeutic E2 accelerated resolution of lung injury in male mice.

**Figure 4:**
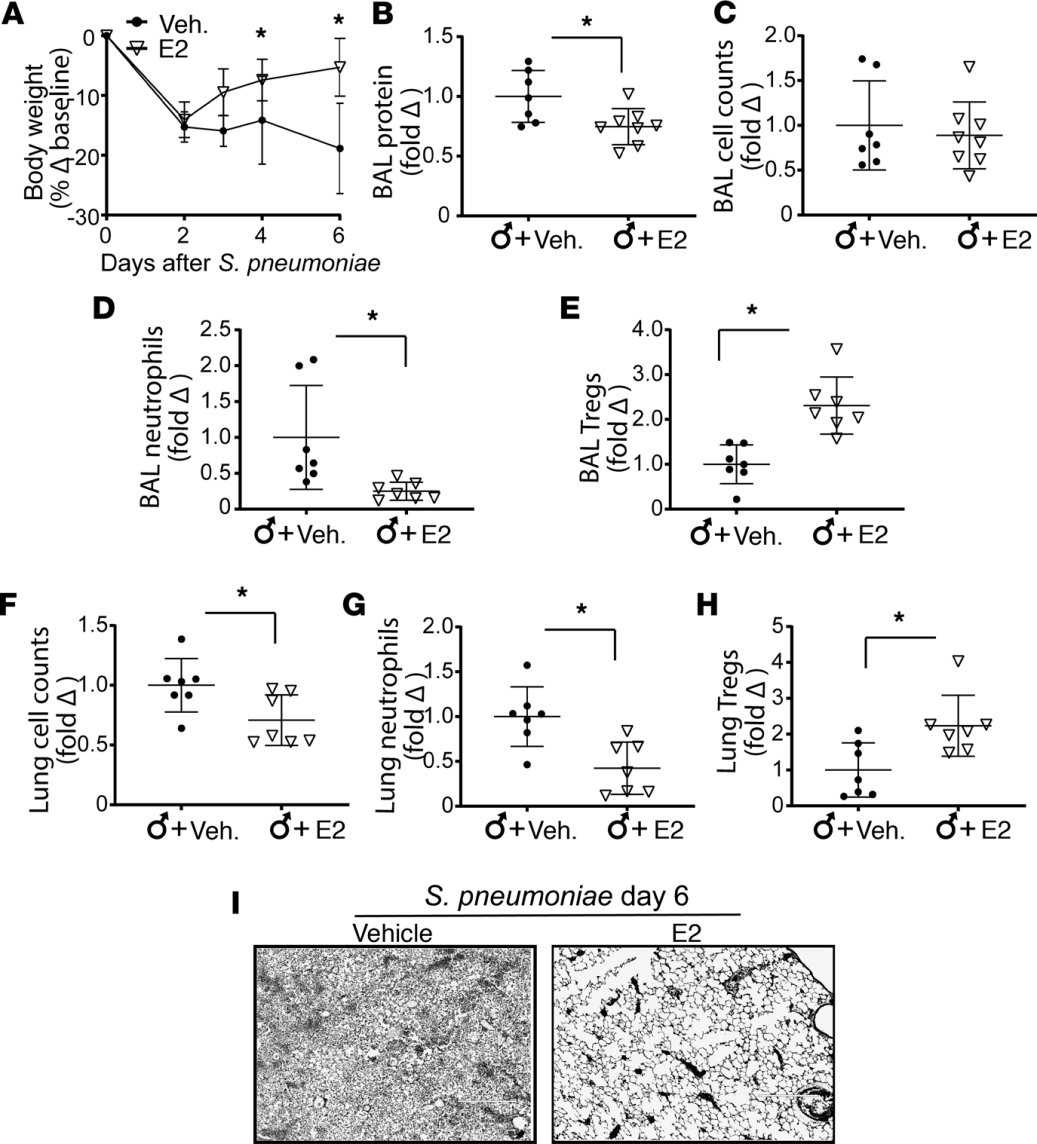
Therapeutic estradiol accelerates resolution of lung injury in male WT animals. Male WT mice were challenged with intratracheal *S*. *pneumoniae* (4 × 10^6^ CFU/mouse). On day 2 after injury, rescue treatment with intraperitoneal estradiol (25 μg/mouse/dose) was administered daily on days 2, 3, and 4. Lung injury markers were measured on day 6 after lung injury. (**A**) Body weight over time relative to baseline at day 0. Fold changes compared with male mice that received intraperitoneal vehicle after *S*. *pneumoniae* were measured for BAL total protein (**B**), BAL total cell counts (**C**), BAL neutrophil counts (**D**), BAL Treg numbers (**E**), lung total cell counts (**F**), lung neutrophils (**G**) and lung Tregs (**H**) are shown. (**I**) Representative lung H&E sections 6 days after injury were stained with H&E. Original magnification, ×100. Two-way ANOVA was used for weight. Normalization to fold change followed by Mann-Whitney test was used for protein and cell counts. *n* = 7 per group. *P < 0.05.

We hypothesized that exogenous E2 could promote the resolution of ALI in male mice given the favorable phenotype observed in female mice (Figure 1) and the enhanced Treg phenotype seen in vitro (Figure 3). To avoid potentially blunting the initial inflammatory response to *S*. *pneumoniae*, we started rescue treatment with E2 at day 2 after lung injury. Male mice treated with vehicle group sustained weight loss, whereas E2-treated male mice regained weight (Figure 4A). At day 6 after lung injury, E2-treated male mice, but not vehicle-treated mice, displayed a resolving phenotype similar to that of female mice, with reduced BAL protein (Figure 4B), decreased BAL neutrophils counts (Figure 4D), and increased BAL Treg numbers (Figure 4E). Total BAL cell count was not statistically different following E2 therapy (Figure 4C), while lung cell counts were diminished (Figure 4F). The lung profile paralleled alveolar compartment profile with decreased lung inflammatory cells, inflammatory cytokines (Supplemental Figure 7), and increased lung Tregs (Figure 4, G and H). Similar to that in female mice, the proportion of Tregs in alveolar and lung compartments of E2-treated mice was increased (Supplemental Figure 6). Moreover, E2-treated male mice displayed higher Ki-67 expression in their Tregs (Supplemental Figure 6), indicating a higher proliferative state. Analysis of BAL cytokines demonstrated that systemic exogenous E2 in male mice reduced BAL proinflammatory cytokines, including IFN-γ, IL-12, IL-6, TNF-α, and IL-1β, without influencing KC, IL-10, or IL-4 levels (Supplemental Figure 7). Representative lung H&E sections showed clearance of lung inflammation in the male E2-treated group (Figure 4I). In summary, rescue therapeutic administration of E2 promoted resolution of ALI in male mice associated with decreased inflammatory cytokine production and increased the number and proliferation of lung Tregs.

### Rescue E2 did not impact lung bacterial clearance.

**Figure 5:**
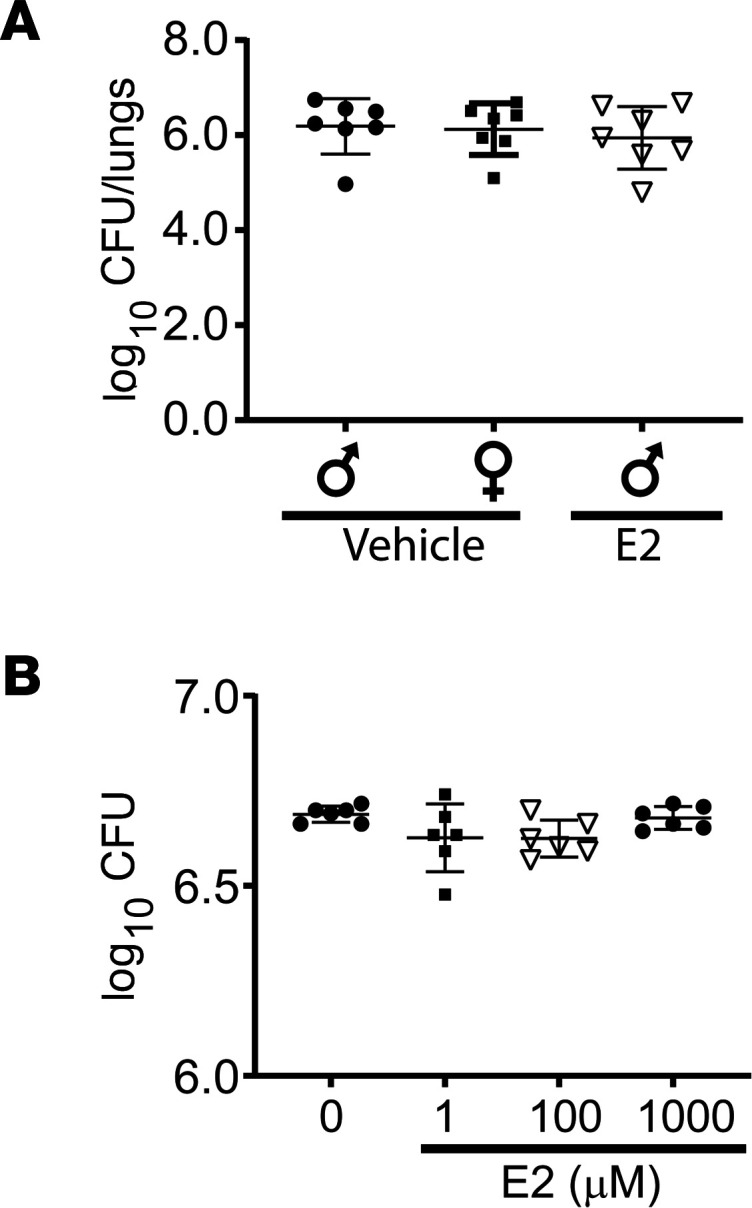
Estradiol does not alter lung bacterial counts after *S*. ***pneumoniae*.** (**A**) Lung homogenates were obtained from WT male and female mice after intratracheal *S*. *pneumoniae* (4 × 10^6^ CFU/mouse) on day 6, plated in blood agar plates overnight at 37°C, and counted for CFU. (**B**) To determine if estradiol has direct antibacterial effects, *S*. *pneumoniae* was plated with vehicle or 1, 100, or 1000 μM E2 overnight in blood ager plates, and CFU were counted. Mann-Whitney test was used. *n* = 7 per group. Values are reported are mean ± SEM.

A potential explanation for improved resolution as a function of sex or mediated by exogenous estrogen is enhanced lung bacterial clearance. In order to investigate both sex differences and whether exogenous E2 had an effect on *S*. *pneumoniae* clearance during resolution. We injected exogenous E2 or vehicle on days 2–4 after lung injury. On day 6 after injury, lungs were harvested and homogenized for determination of colony CFU for *S*. *pneumoniae*. Male and female mice had no demonstrable difference in bacterial loads, and E2 treatment of male mice showed no difference in bacterial load (Figure 5A). Moreover, to determine whether E2 exhibited direct bactericidal activity, we cultured *S*. *pneumoniae* in the presence of increasing concentrations of E2 (1–1000 μM) or vehicle. After culturing for 24 hours, CFU were counted. We observed no difference in CFU between E2 and vehicle-treated *S*. *pneumoniae*, suggesting a lack of direct bactericidal activity by E2 (Figure 5B). These studies suggest that sex differences in PNA outcomes and the E2 therapeutic effects were unlikely to be due to modulation of lung bacterial burden.

### Tregs are required for E2 enhanced resolution.

**Figure 6:**
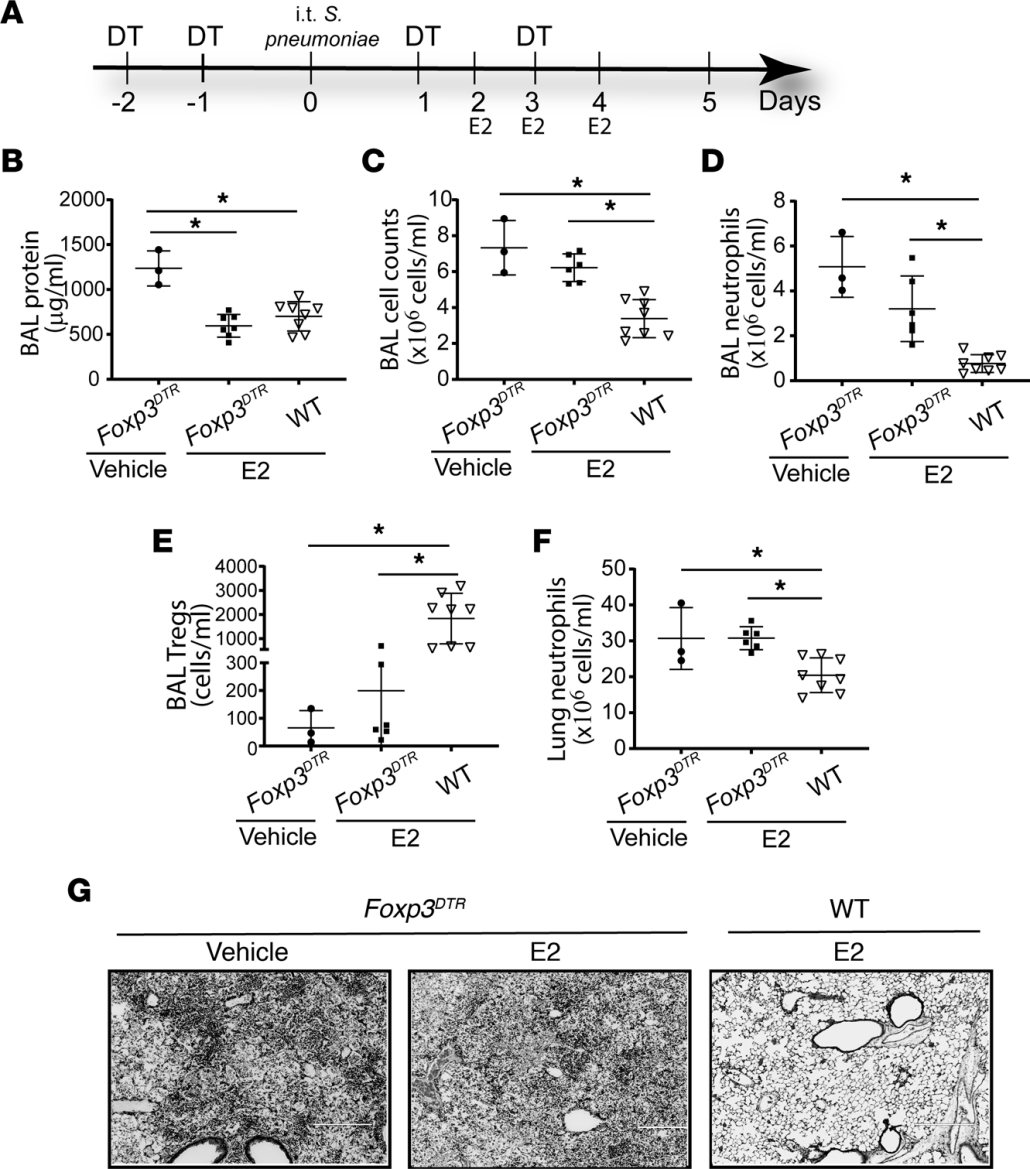
Salutary effects of E2 require Tregs. Male WT and *Foxp3^DTR^* mice were challenged with intratracheal *S*. *pneumoniae* (3 × 10^6^ CFU/mouse). All groups received diphtheria toxin (day –2 at 50 μg/kg, subsequent doses at 10 μg/kg), and estradiol (E2; 25 μg/mouse/dose) treatment was given intraperitoneally daily on days 2, 3, and 4, as shown in the schematic (**A**). Lung injury markers were measured on day 5 after injury. BAL total protein (**B**), BAL total cell counts (**C**), BAL neutrophil counts (**D**), BAL Tregs (**E**), and lung neutrophils (**F**) were measured 6 days after *S*. *pneumoniae* injury. (**G**) Representative lung sections were stained with H&E. Original magnification, ×100. One-way ANOVA was used. *n* = 3–8 per group. *P < 0.05. Values are reported are mean ± SEM.

In order to determine if the salutary effects of E2 required Tregs in vivo, we treated *Foxp3^DTR^* mice with exogenous diphtheria, efficiently depleting Tregs. Male *Foxp3^DTR^* mice and age-matched WT counterparts received diphtheria toxin beginning 2 days before *S*. *pneumoniae* injury and every other day thereafter. E2 was given intraperitoneally daily starting on days 2–4 (Figure 6A). We found that Tregs were critical to resolve *S. pneumoniae* (Supplemental Figure 8). We confirmed lung Treg depletion in diphtheria toxin–treated *Foxp3^DTR^* mice compared with WT mice 5 days after *S*. *pneumoniae* injury (Supplemental Figure 9). In contrast to the beneficial effects of E2 in injured WT mice, E2 treatment in Treg-depleted *Foxp3^DTR^* mice did not accelerate lung injury resolution, as shown by persistent, elevated BAL total cell counts (Figure 6C), BAL neutrophils (Figure 6D), lung neutrophils (Figure 6F), and histological changes (Figure 6G). Treg levels measured in the BAL were significantly higher in Treg-sufficient WT mice treated with E2 (Figure 6E). Lung H&E sections showed persistent injury in the Treg-depleted hosts, despite E2 therapy. In contrast, WT Treg-sufficient animals that received E2 displayed enhanced resolution (Figure 6G). Interestingly, compared with vehicle-treated *Foxp3^DTR^* mice, E2-treated *Foxp3^DTR^* mice showed lower BAL protein levels (Figure 6B), suggesting potential additional Treg-independent mechanisms in E2-medicated lung inflammation recovery. These data confirmed that E2-enhanced resolution of lung inflammation required Tregs.

### E2 enhanced Treg-suppressive phenotype required ERβ.

**Figure 7:**
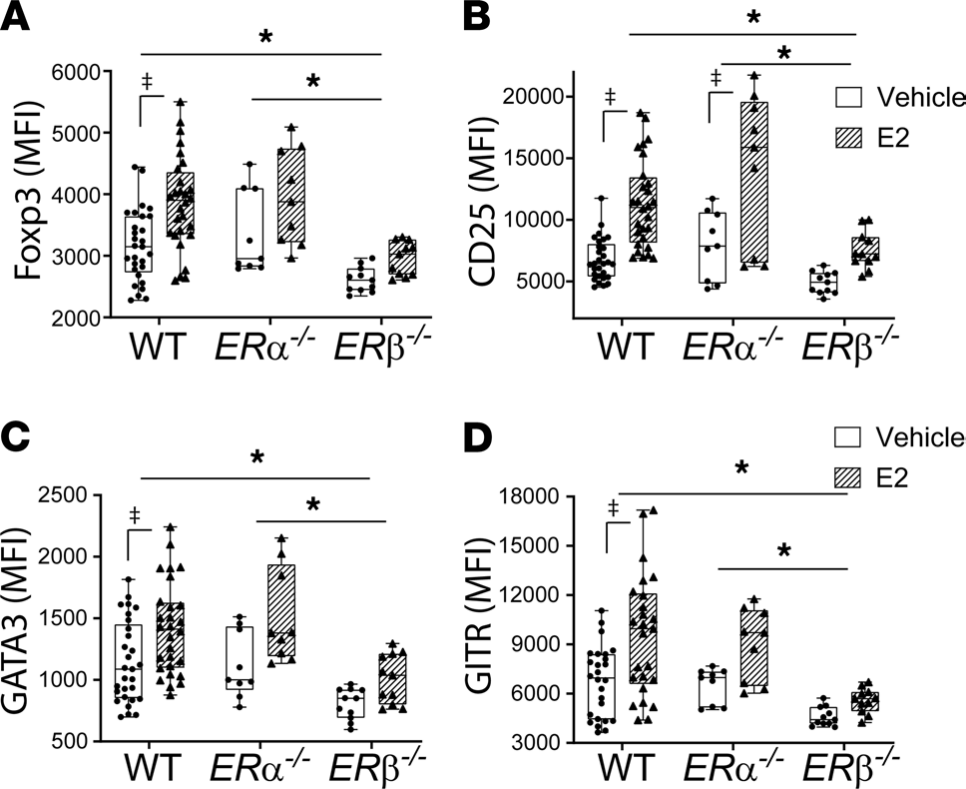
Estradiol augmentation of Treg-suppressive phenotype is ERβ dependent. CD4^+^CD25^+^ Tregs were isolated from male WT, *ERα^–/–^*, and *ERβ^–/–^* splenocytes, cultured in the presence of anti-CD3/CD28 beads, and stimulated with either vehicle or estradiol (E2; 10 μM) for 72 hours. Multicolor flow cytometry was performed for the expression of Foxp3 (**A**), CD25 (**B**), GATA3 (**C**), and GITR (**D**) and measured by mean fluorescence intensity (MFI). Two-way ANOVA was used for statistics. *n* = 9–29 per group. *P < 0.05 for strain interaction response to E2; ‡*P* < 0.05 for E2 responses within a strain. Values are reported are mean ± SEM.

The predominant effects of E2 are mediated through 2 distinct estrogen receptors (ERs), ERα and ERβ. ERα and ERβ signaling can display redundant effects in vitro; however, distinct expression patterns in cells and tissues have shown diverse biological effects in vivo. In order to evaluate the receptor requirement for a Treg-suppressive phenotype, Tregs isolated from WT, *ERα^–/–^*, and *ERβ^–/–^* mice were cultured with anti-CD3/CD28 beads with or without E2 in vitro. Similar to the in vivo findings, E2 induced Foxp3 expression in both WT and *ERα^–/–^* Tregs. In contrast, E2 did not induce Foxp3 expression in *ERβ^–/–^* Tregs (Figure 7A). *ERα^–/–^* Tregs responded to E2 similarly to WT Tregs, with increased Foxp3, GATA3, CD25, and GITR, whereas *ERβ^–/–^* Tregs were unresponsive (Figure 7, A–D, respectively). This hyporesponsive phenotype was not generalized, as *ERβ^–/–^* Tregs responded to exogenous IL-2 in vitro (Supplemental Figure 10). In summary, ERβ was required for E2 to exert its effect on Tregs in vitro.

### E2 prorepair function required ERβ to resolve PNA.

**Figure 8:**
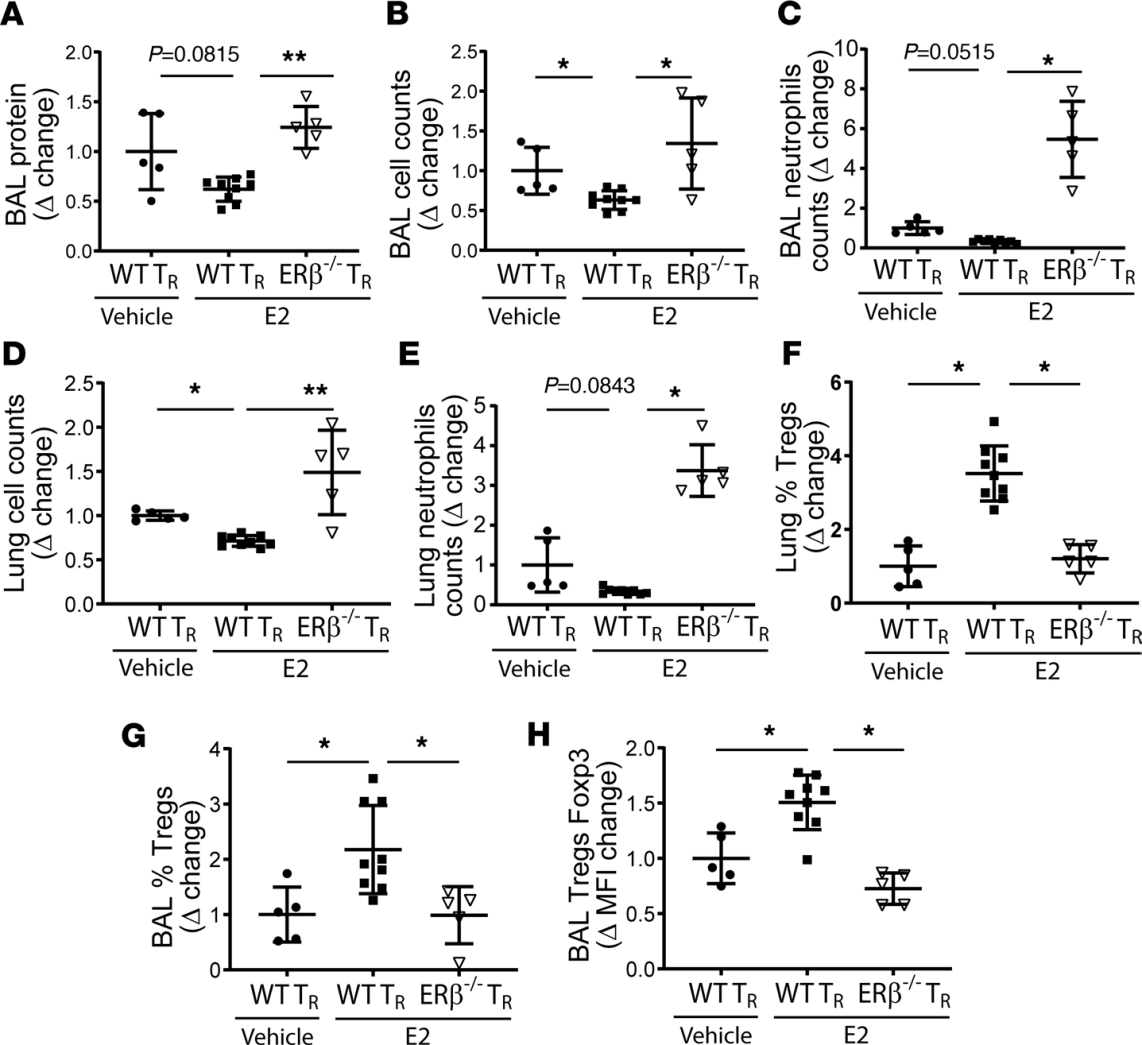
Estradiol augments Treg function in an ERβ-dependent manner. Male WT and *ERβ^–/–^* Tregs were cultured in the presence of anti-CD3/CD28 beads and stimulated with either vehicle or estradiol (E2; 10 μM) for 48 hours. Cells were collected, and 0.25 × 10^6^ Tregs were adoptively transferred (AT; retro-orbital) 1 hour after intratracheal *S*. *pneumoniae* (3 × 10^6^ CFU/mouse) in lymphocyte-deficient *Rag-1^–/–^* mice. Lung injury markers were measured at day 5 and are expressed as fold change compared with *Rag-1^–/–^* mice AT with WT Tregs cultured ex vivo with vehicle (ethanol). BAL protein (**A**), BAL total cell counts (**B**), BAL neutrophil counts (**C**), lung total cell counts (**D**), lung neutrophil counts (**E**), percentage of Tregs in total lung cells (**F**), percentage of Tregs in total BAL cells (**G**), and their relative Treg Foxp3 expression (**H**) were measured. Normalization followed by Kruskal-Wallis test was used. *n* = 5–8. *P < 0.05. Values are reported are mean ± SEM.

To address the contribution of ERβ to E2-medicated effects on Treg biology, we evaluated ex vivo E2-treated Tregs in *S*. *pneumoniae–*induced ALI. Previously, we demonstrated that the therapeutic adoptive transfer of Tregs requires 1 × 10^6^ cells/mouse and that transferring lower numbers would be insufficient to mediate robust resolution (34). *S*. *pneumoniae*–injured lymphocyte-deficient *Rag-1^–/–^* mice received 0.25 × 10^6^ WT vehicle-treated Tregs or E2-treated WT or *ERβ^–/–^* Tregs via retro-orbital injection 1 hour after intratracheal *S*. *pneumoniae*. Five days after injury, mice that received a subtherapeutic dose of E2-treated *ERβ^–/–^* Tregs displayed unremitting lung inflammation characterized by elevated BAL protein (Figure 8A), high BAL total cell counts (Figure 8B), and high BAL neutrophil counts (Figure 8C and Supplemental Figure 11) and total lung neutrophil counts (Figure 8E and Supplemental Figure 11) as well as total lung cell counts (Figure 8D). Thus, a nonresolving lung inflammatory phenotype was observed in *Rag-1^–/–^* mice that received E2-treated *ERβ^–/–^* Tregs. In contrast, lung injury resolution occurred in mice that received E2-treated WT Tregs (Figure 8, A–F). Flow cytometry confirmed successful adoptive transfer and homing to the alveoli and lungs independent of ERβ expression (Figure 8, F and G, and Supplemental Figure 10). Interestingly, the absolute numbers of recovered adoptively transfer Tregs did not differ among groups (Supplemental Figure 10), although E2-treated WT Tregs displayed higher percentages of BAL Tregs and Foxp3 expression (Figure 8, G and H). In contrast, transfer of E2-treated *ERβ^–/–^* Tregs resulted in higher BAL and total lung cellularity and BAL neutrophil counts as well as loss of E2-mediated increases in the proportion of BAL Tregs. Analysis of Treg phenotypic markers indicated that *ERβ^–/–^* Tregs treated with E2 had similar expression of proliferative markers Ki67 but diminished expression of CD39 (Supplemental Figure 12). This, coupled with diminished expression of Foxp3 and insufficient rescue in *Rag-1^–/–^* mice, indicated that *ERβ^–/–^* Tregs have reduced suppressive phenotype and function. In summary, ex vivo treatment of an otherwise ineffective Treg dose facilitated lung injury resolution and Treg expression of ERβ was necessary for Treg-mediated resolution of *S*. *pneumoniae–*lung injury. Tregs were gated using a conventional gating strategy (Supplemental Figure 13).

### Tregs modulate macrophages responses via E2/ERβ.

**Figure 9:**
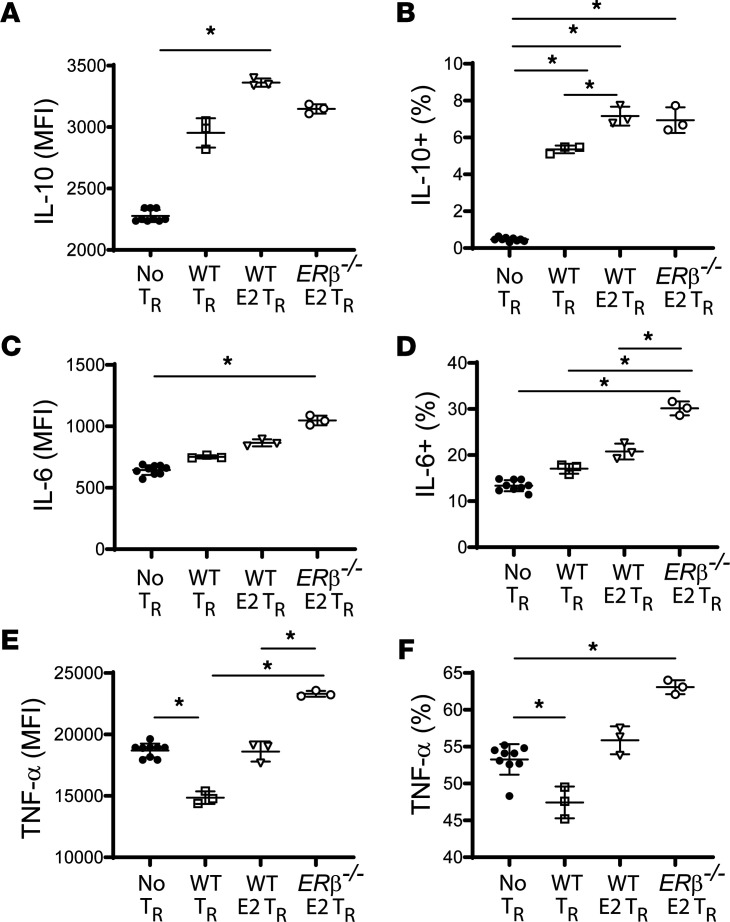
Tregs modulate macrophages responses via E2/ERβ. WT and *ERβ^–/–^* Tregs were isolated and cultured for 48 hours with CD23/CD28 dynabeads in the presence or absence of estradiol (10 μM). Tregs were harvested, counted, and transferred to wells containing LPS-stimulated bone marrow–derived macrophages at a lymphocyte-to-macrophage ratio of 1:2 for 24 hours. During the last 4 hours of coculture, brefeldin A was added. Coculture cells were harvested for intracellular flow cytometry. Macrophage IL-10 (**A** and **B**), IL-6 (**C** and **D**), and TNF-α (**E** and **F**) were measured as mean fluorescence intensity (MFI) and as percentage positivity from total macrophage subpopulation (CD4^–^ population). One-way ANOVA was used. *n* = 3–8 per group. *P < 0.05. Values are reported are mean ± SEM.

To begin to understand the cellular effector targets mediating the salutary effects of E2/ERβ in Tregs, we cocultured macrophages with Tregs. Tregs were isolated from WT or *ERβ^–/–^* spleens and cultured for 48 hours in the presence or absence of E2 (10^-5^ M). Pretreated Tregs were then transferred into direct contact with LPS-stimulated (100 ng/ml) bone marrow–derived macrophages (BMDMs) at a ratio of 1:2 (lymphocyte/macrophage). After 24 hours in cocultures, cells were harvested for intracellular cytokine staining for macrophage-derived cytokines. Nonprimed WT Tregs abrogated macrophage TNF-α while increasing antiinflammatory IL-10 production (Figure 9, A and E). E2-primed WT Tregs surprisingly did not abrogate macrophage TNF-α responses, but rather further increased their IL-10 production compared with nonprimed WT Tregs (Figure 9A). In contrast, E2-primed ERβ^–/–^ Tregs exacerbated macrophage proinflammatory responses with higher BMDM IL-6 and TNF-α production (Figure 9, C–F), while IL-10 production was still enhanced compared with coculture with nonprimed WT Tregs (Figure 9, A and B). These findings support an E2/ERβ role for Tregs to modulate macrophages inflammatory responses.

## Discussion

We reported therapeutic efficacy for exogenous E2 in promoting resolution of experimental PNA-ALI. Our findings supported that E2-mediated beneficial effects were dependent on CD4^+^CD25^+^Foxp3^+^ (Tregs) cells. E2 enhanced the Treg prorepair phenotype and function to mediate and accelerate resolution of lung inflammation induced by PNA. The E2 modulation was dependent on Tregs and expression of ERβ and independent on antibacterial properties.

Although there is no ideal model that recapitulates the complex underlying mechanisms of human ALI (39), we chose a direct model of PNA with *S*. *pneumoniae*. *S*. *pneumoniae* is one of the leading causes of PNA worldwide (40). It produces a robust initial lung inflammatory response that is reproducible and a resolution phase that can be evaluated over time. We treated animals with E2 starting at day 2 after initial injury for several reasons. First, we focused on the resolution phase, a distinct stage with active immunological mechanisms (41, 42) that could offer new therapeutic targets. Second, pretreatment or early delivery of E2 could blunt the peak inflammatory injury and thus hasten resolution of inflammation. Finally, and most importantly, patients often present days after their onset of PNA, and thus, assessing delayed treatment (i.e., rescue) effects provides a more clinically relevant therapeutic model. Systemic fluids and antibiotics, cornerstone treatments for PNA, were not used in our studies to avoid confounding variables, although they could be used in future studies using more severe models of lung infections, conditions which could necessitate multiple interventions strategies.

The burden of infectious diseases is generally higher in men than in women (6, 10). Preclinical models of lung inflammation have demonstrated a protective effect of females over males (17, 43–46); however, there has been a lack of cellular/molecular mechanisms that provide an explanation for the female salutary effects. Our studies support the role of sex as a major determinant in resolution of pneumococcal PNA. Compared with their male counterparts, female mice exhibited enhanced resolution of severe pneumococcal PNA, with decreased lung inflammation and enhanced clearance of alveolar and lung neutrophils. This was independent of effects on bacterial clearance. In contrast to reports using pretreatment with E2 (18, 20), resolution can be accelerated by E2 administered 2 days after established lung injury. The therapeutic treatment of male mice with E2 had no effect on lung bacterial load clearance, indicating that E2 was not bactericidal and did not affect bacterial burden, but rather targets enhanced prorepair mechanisms.

Therapeutic effects of E2 have been reported in models of carrageenan-induced lung injury (47) and models of sepsis induced by cecal ligation and puncture (48). These studies used either preventive or early treatment strategies, limiting their clinical translation. We chose rescue therapeutic administration of E2 in order to minimize the potential effect on early beneficial inflammatory responses in the lung early in bacterial lung infection.

E2 also modulates macrophage responses and reprograms them to alternative activated and antiinflammatory states (49–51). Most bacterial infections induce classically activated macrophages, which are important for initially clearance of infections and subsequent skewing to a prorepair state that promotes healing. Our experiments using Treg-depleted animals suggest that E2-medicated prorepair effects were independent of direct E2 effects on macrophages but support a model in which E2-treated Tregs modify macrophage responses. In the normal host, E2 could contribute via modulation of macrophages to an alternative activated state and thus promote Treg numbers and their suppressive phenotype (52, 53). We also observed decreased BAL protein in Treg-depleted animals that received E2. This effect suggests that nonimmune cells, such as endothelial cells, could also be relevant targets of E2 in vivo. E2 regulates vascular inflammation with antiinflammatory effects through direct antioxidant effects, generation of nitric oxide, reduction of endothelial cell apoptosis, and suppression of cytokines (54). Although E2 administration has potent effects in multiple cells types, our studies support a requirement for Tregs to mediate E2 salutary effects in resolution of pneumococcal-induced ALI.

Resolution of lung injury is an active process. Tregs maintain immunological self-tolerance and homeostasis by suppressing aberrant or excessive immune responses harmful to the host (27). We showed that CD4^+^CD25^+^Foxp3^+^ Tregs resolve experimental ALI by modulating critical prorepair steps: (a) abrogation of macrophage proinflammatory responses, (b) augmentation of neutrophil efferocytosis (29), (c) limitation of fibroproliferation (30), and (d) augmentation of alveolar epithelial repair (31). In this report, we determined the feasibility of using E2 to promote Treg function in vivo and ex vivo to improve PNA-ALI outcomes.

We have observed that female mice had higher BAL and lung Treg numbers, with higher levels of Foxp3 and Ki-67 expression (a marker of proliferation) after injury, indicating that sex hormones could enhance the suppressive function and proliferative rate of Tregs during resolution. Although there are several sex hormonal differences in females compared with males, we focused on E2 given reports of its effect on Tregs (36, 55–57). Arruvito et al. described a positive correlation of E2 levels with Treg numbers in women (58). Polanczyk et al. demonstrated that E2 treatment of isolated CD4^+^ splenocytes increased their CD25 protein expression and induced FOXP3 mRNA (55). They showed enhanced suppressive activity of Tregs isolated from E2-treated mice when coculture with T effector cells.

In order to evaluate the E2 effects on Treg-suppressive phenotype, we performed an extensive survey of Treg proteins using multicolor flow cytometry. Our immunophenotyping evaluation of Tregs suggested several mechanisms involved in E2-enhanced lung repair. First, E2 augmented the expression of Treg master transcription factor, Foxp3. Enhanced Foxp3 correlates with higher immunoregulatory and suppressive function (59). Interestingly, E2 induced Foxp3 expression in Tregs but not in CD4^+^CD25^–^ cells. This is in contrast to a previous report showing that E2 promoted the conversion of CD4^+^CD25^–^ T cells to CD4^+^CD25^+^ T cells. Tai et al. showed a subtle increase in Foxp3 expression in CD4^+^CD25^–^ T cells treated with E2, from 1% of to 3% in total CD4^+^ cells in a representative sample (57). Second, we also found another critical Treg transcription factor, GATA3, regulated by E2. GATA3 is essential for the homeostasis and stability of Tregs. GATA3 is required to maintain high levels of Foxp3 and CD25 expression in inflammatory sites (60, 61). We observed different effects of E2 on Treg GATA3 expression, with increased expression in vitro that was unchanged in vivo. The expression of GATA3 in Tregs can be negatively regulated by a number of inflammatory cytokines, including IL-6, IL-27, and IL-12. These inflammatory cytokines may contribute to the decreased GATA3 expression observed in the inflamed hosts (62–65), while in vitro Treg stimulation with E2 significantly increased GATA3 expression. To our knowledge, the modulation of E2 on Treg GATA3 expression has not previously been reported. Third, GITR was induced by E2. GITR is expressed at high levels in activated T cells and Tregs. Although GITR is not essential for Treg-suppressive function in vitro (66), GITR has an important role in Treg expansion (67), with lower number of Tregs seen in GITR-knockout mice (68, 69). GITR activation on Tregs can exert distinct roles based on which signaling pathways are activated. The role of Treg GITR is also dependent on the experimental context (homeostatic vs. inflammatory). For instance, activation of the NF-κB pathway may result in Treg expansion (67), while Siva protein activation may result in Treg apoptosis (70). The specific role of E2 induction of Treg GITR expression will need to be further investigated in context of lung inflammation.

In summary, E2 increased several critical components of Treg function, and the synergistic effect of this upregulation in Treg markers likely led to an enhanced Treg function. Collectively, both quantitative factors (augmentation of Treg-suppressive function) and qualitative factors (increased Treg proliferation and numbers) may underlie the proresolution effect of E2. E2 functions primarily through 2 intracellular ERs (ERα and ERβ) (71). These receptors are ubiquitously expressed (72), with a wide range of ER expression across tissues, remarkably, with few sex differences (73). Other reports indicate that ERα is necessary for E2-mediated upregulation of intracellular PD-1 expression in Tregs, contributing to its suppressive function (56). In contrast, we found that ERα was not required for therapeutic E2-mediated augmentation of Treg-suppressive phenotype and function. In contrast, Treg expression of ERβ was necessary for E2-mediated resolution of pneumococcal ALI and phenotypic changes of Tregs in vitro.

The contribution of ERα and ERβ to the E2-mediated effect may vary based on the source of the Tregs. An E2-mediated effect on induced Tregs (iTregs) has been reported, showing that specific depletion of ERα on total CD4^+^ cells attenuated the E2 response of iTregs (52). We focused on thymic-derived Tregs and not iTregs. Although we cannot exclude a relative contribution of iTregs, our adoptive transfer experiments exclusively used Tregs. *ERα^–/–^* Tregs stimulated with E2 upregulated Foxp3, GATA3, CD25, and GITR expression in a similar manner to WT Tregs. In contrast, *ERβ^–/–^* Tregs failed to upregulate this “estrogenic signature.” This was not a sign of global hyporesponsiveness in vitro. *ERβ^–/–^* Tregs treated with IL-2 showed a robust increase in Foxp3 and CD25 expression, confirming that the hyporesponsive state was specific for E2. Individuals with multiple sclerosis have lower expression ERβ in Tregs compared with controls (74), underscoring the potential importance of ERβ expression in Tregs in humans.

Tregs orchestrate resolution of lung inflammation and promote lung repair through cellular interactions with macrophages (29), alveolar epithelial cells (31), and, likely, endothelial cells. Our findings suggest that E2-treated Tregs enhance the production of macrophage IL-10, while having no additive effects in downregulating macrophage proinflammatory responses. These findings were ERβ independent. However, although E2-treated *ERβ^–/–^* Tregs were able to augment macrophage IL-10 production, their effect on macrophage proinflammatory responses was significantly augmented, elucidating the enhanced alveolar inflammatory milieu observed in our adoptive transfer experiments with E2-treated *ERβ^–/–^* Tregs (Figure 8). E2/ERβ signaling in Tregs could be responsible for Treg lineage commitment and maintenance of Foxp3 expression (75), and a lack of it could render these cells into ex-Foxp3 Tregs with a promiscuous and proinflammatory effect (e.g., Th1 or Th17). Treg lineage tracing experiments will be needed to evaluate this hypothesis. We did not observe Tregs enhancing macrophage TGF-β in our coculture experiments, a finding we had previously described (29). These coculture experiments were different from previous experiments, as Tregs were cultured for 48 hours and maximally stimulated before their coculture with stimulated macrophages. E2/ERβ signaling in Tregs and their prorepair effects on other immune and nonimmune injured cells will need the focus of future studies. Moreover, the transcriptional and proresolution signatures induced by E2/ERβ signaling in Tregs will yield valuable information and provide other targets for resolution of PNA.

The present study has limitations and raises questions. What other cell types are modulated in response to E2 in the setting of lung injury resolution? A recent investigation showed that E2 inhibited the LPS-induced IL-6 inflammatory response, resulting in inhibition of NF-κB transcriptional activity via GPR30/GPER1 in monocytes (76). Yang et al. reported that estrogen-mediated activation of lung macrophage nitric oxide synthase-3 was involved in female resistance to PNA (22). Our studies do not directly evaluate whether physiological levels of E2 were sufficient to mediate its prorepair effects. E2 can display different effects on human monocytes/macrophages, with low doses enhancing the production of proinflammatory cytokines and high doses reducing their production (15). We also did not address if androgens or other sex hormones modulate Tregs during PNA resolution. Androgens and progesterone have been reported to increase the Treg population and Foxp3 expression (77–79). Our studies focused on the therapeutic implication of exogenous E2 and did not systematically define alternative determinants for sex differences in the resolution of PNA lung injury.

We believe our findings have translational relevance to PNA-ALI. Although systemic administration of E2 represents a potential therapeutic strategy, ex vivo treatment of Tregs with E2 followed by cell transfer could improve E2’s therapeutic index. Tregs could be sorted from individuals with severe PNA and ex vivo primed and stimulated with E2 (24–48 hours of stimulation) with subsequent transfer back to the host (37). We have shown the feasibility of this approach (80), and others have suggested it as a potential therapeutic strategy for Treg immunotherapy (38, 81, 82).

In conclusion, we reported a role for rescue treatment with E2 in the resolution of PNA. Tregs were indispensable for the resolution of PNA. Moreover, E2 prorepair effects required Tregs and specifically ERβ expression. We hope to provide the foundation for nonantibiotic therapeutic targets for PNA-induced lung injury and potential consideration of cellular therapy with “conditioned” Tregs.

## Methods

### Animals.

C57BL/6 WT, *Rag-1^–/–^*, *ERα^–/–^*, and *ERβ^–/–^* mice were purchased from The Jackson Laboratory. *Foxp3^DTR^* mice were a gift from Alexander Rudensky (Sloan-Kettering Institute, New York, New York, USA). Animals were bred and housed in a pathogen-free facility. Mice aged 8–12 weeks were used. Male mice were age matched with counterpart female mice. Both female and male mice were harvested simultaneously at specified time intervals after infection.

### *S*. *pneumoniae* preparation.

*S*. *pneumoniae* (serotype 19, ATCC 49619) was purchased ATCC. Bacteria were grown overnight at 37°C in a 5% CO_2_ incubator on blood agar plates with 5% sheep blood in tryptic soy agar (Thermo Fisher Scientific). About 10 colonies were then suspended in Todd-Hewitt Broth (BD) supplemented with 17% (v/v) Fetal Bovine Serum (Thermo Fisher Scientific) and incubated at 37°C with shaking at 225 rpm for 4–6 hours until an OD_600_ 0.3 was reached. The media were distributed into 1 mL aliquots and flash frozen in liquid nitrogen before storage at –80°C. Freshly thawed aliquot was used to challenge mice and subsequently plated to confirm the CFU instilled.

### Preparation of mice.

Mice were anesthetized with intraperitoneal ketamine/acetylpromazine (100 and 2.5 mg/kg, respectively) prior to intubation with a 20-gauge catheter. After anesthesia and tracheal intubation, *S*. *pneumoniae* or Todd Hewitt broth (RPI, T47500) was injected into the trachea. 25 μg β-Estradiol (TOCRIS Biosciences) or vehicle was given on days 2–4 through intraperitoneal injection after inoculation. For Treg depletion, diphtheria toxin (List Biologicals) was administered intraperitoneally 2 days and 1 day before intratracheal instillation of bacteria and then on days 1 and 3 after inoculation. At day 5 or 6 after *S*. *pneumoniae* instillation, mice were anesthetized and killed by isoflurane (Fluriso, MWI).

### Analysis of BAL fluid.

BAL was obtained by cannulating the trachea with a 18-gauge catheter. The bilateral lung was lavaged twice (each aliquot, 1 ml; calcium-free PBS); total returns averaged 1.6–1.8 ml/mouse. BAL was centrifuged at 500*g* for 5 minutes at 4°C. The cell-free supernatants were stored at –80°C for later analysis. The cell pellet was diluted in PBS, and the total cell number was counted with a hemocytometer after staining with trypan blue. Differential counts were done on cytocentrifuge preparations by counting 300 cells per sample (Cytospin 3; Shandon Scientific) and stained with Hema 3 Stat Pack (Fisher HealthCare) according to instructions. Total protein was measured in the cell-free supernatant using the Pierce BCA protein assay kit (Thermo Fisher Scientific).

### Preparation of lung single-cell suspension.

Lungs were gently minced using a MACS Dissociator (Miltenyi Biotec), incubated at 37°C in an enzyme cocktail of RPMI containing 5 mg/ml collagenase I (Worthington) and 1 mg/ml DNase (MilliporeSigma), and then mashed through a 70 μm nylon cell strainer (BD Falcon). Red cells were lysed using ACK lysing buffer (Quality Biological), and then single-cell suspension was obtained.

### Lung morphology.

Lungs from animals were inflated to 25 cm H_2_O with formalin solution (MilliporeSigma) for histologic evaluation by H&E staining as previously described (29).

### Flow cytometry.

BAL cells and single-cell suspensions were prepared for FACS analysis with a live-dead discriminator and ﬂuorochrome-conjugated antibodies. Cells were incubated with Fc Block (BD Biosciences) antibody to block Fcγ III/II receptors before staining with a specific antibody. The following antibodies (BD Biosciences — Pharmingen) were used for surface staining: BUV395-conjugated anti-CD4 (clone GK1.5), BV650-conjugated anti-CD25 (clone PC61), PE-Cy7-conjugated anti-CD39 (clone 24DMS1), APC-Cy7-conjugated anti-CD62L (clone MEL-14), BV510-conjugated anti-CD44 (clone 1M7), BV605-conjugated anti-PD-1 (clone 29F.1A12), BV711-conjugated anti-GITR (clone DTA-1), BUV737-conjugated anti-CD69(clone H1.2F3), and BV786-conjugated anti-CD40L (clone MR1). For intracellular staining, cells were fixed and permeabilized with Foxp3 staining buffer (eBioscience), then stained with APC-conjugated anti-Foxp3 (clone FJK-16s), PE-CF594–conjugated anti-GATA3 (clone L50-823), PerCP-eFlour710–conjugated anti-Ki-67 (clone SoLA15), and BV421-conjugated anti-CTLA-4 (clone UC10-4B9). Lymphocytes were gated with characteristic low forward scatter/side scatter, using a FACSAria instrument and FACSDiva for data acquisition (BD) and Flowjo for analysis (Tree Star Inc.). Mean ﬂuorescence intensity was calculated as the mean of the positive population ﬂuorescence.

### BAL cytokines measurements.

BAL supernatant was collected following centrifugation of the cellular components and stored at –80° until further processing. Cytokine measurements were performed using the Mesoscale Discovery platform.

### Lymphocyte culture.

Splenic CD4^+^CD25^+^ cells (about 85%–90% Foxp3^+^) and CD4^+^CD25^–^ (<1% Foxp3^+^) cells were isolated using magnetic bead separation (Miltenyi Biotec). Cells were plated in media with Mouse T-cell activator CD3/CD28 Dynabeads (ratio 1:1; Gibco). Cells were incubated for 72 hours with vehicle or E2 at 10 μM. Cells were then stained for flow cytometry.

### Adoptive transfer.

CD4^+^CD25^+^ isolated from male WT or *ERβ^–/–^* splenocytes were cultured with vehicle or 10 μM E2 for 48 hours as above. Cells were spun down and resuspended in 100 μl PBS. 0.25 × 10^6^ live Tregs were administered via retro-orbital injection into male *Rag-1^–/–^* mice 1 hour after intratracheal *S*. *pneumoniae*.

### BMDMs.

BMDMs were isolated from WT C57BL/6 femurs by flushing with 10 ml RPMI medium using a 27-gauge needle into 6-well plates. Single-cell suspensions were centrifuged (300*g*) and resuspended in RPMI medium containing M-CSF (40 ng/ml) and plated for a total of 7 days before cocultures.

### Macrophage-Treg cocultures.

Isolated Tregs were cultured for 48 hours with CD23/CD28 dynabeads in the presence or absence of E2 (10 μM). Cells were harvested, counted, and transfered to wells containing LPS-stimulated BMDMs at a lymphocyte-to-macrophage ratio of 1:2 for 24 hours. At 20 hours, brefeldin A was added for intracellular cytokine staining.

### Statistics.

Groups of 3–7 mice were used for all experiments, and experiments were repeated at least twice. In vitro experiments were performed in triplicate and repeated at least 3 times. Values are reported as mean ± SEM. Differences between groups were compared using Mann-Whitney *U* test. Multiple group comparisons were performed using 1-way ANOVA with Tukey’s multiple comparisons test. Two-factor comparisons were performed using 2-way ANOVA Tukey’s multiple comparisons test. Significance was determined at *P* values of less than 0.05. Statistics were performed using GraphPad Prism or R (83).

### Study approval.

The Institutional Animal Care and Use Committee of the Johns Hopkins University School of Medicine approved the animal procedures performed in this study.

## Author contributions

YX and TP carried out the experiments. QZ provided technical expertise. AB and LW acquired data. KS analyzed data. YX and FRD wrote the manuscript. RD and KS provided intellectual input. FRD, RD, and YX designed and supervised the study. All authors read and approved the final manuscript.

## Supplementary Material

Supplemental data

## Version 1 12/08/2020

In-Press Preview

## Version 2 02/08/2021

Electronic publication
