# Supplementary material for: Estradiol resolves pneumonia via ERβ in regulatory T cells
Source: JCI Insight. 2021 Feb 8;6(3):e133251. doi: 10.1172/jci.insight.133251 (PMC7934849; doi:10.1172/jci.insight.133251)
Supplement: Supplemental data [file jciinsight-6-133251-s169.pdf]

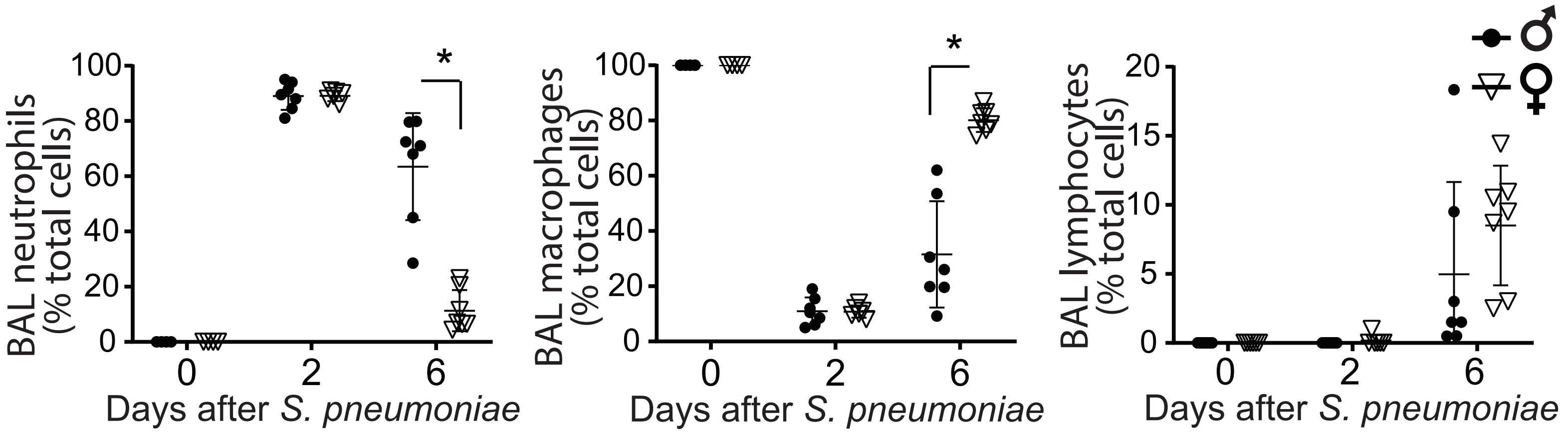

Supplementary Fig. E1.

Female mice displayed with enhanced resolving parameters of pneumonia. Age-matched WT male and female mice were challenged with intratracheal *S. pneumoniae* (4 x 10^6 CFU/mouse) and followed over time. Lung injury parameters were harvested on days 0, 2 and 6. The percentage of BAL neutrophils, BAL macrophages and lymphocytes were determined over time in female and male WT mice after intratracheal *S. pneumoniae* by cytospin. Two-way ANOVA was used for statistics. Mice, n=6-7 per group per time point. *P <0.05. Values reported as mean ± SEM.

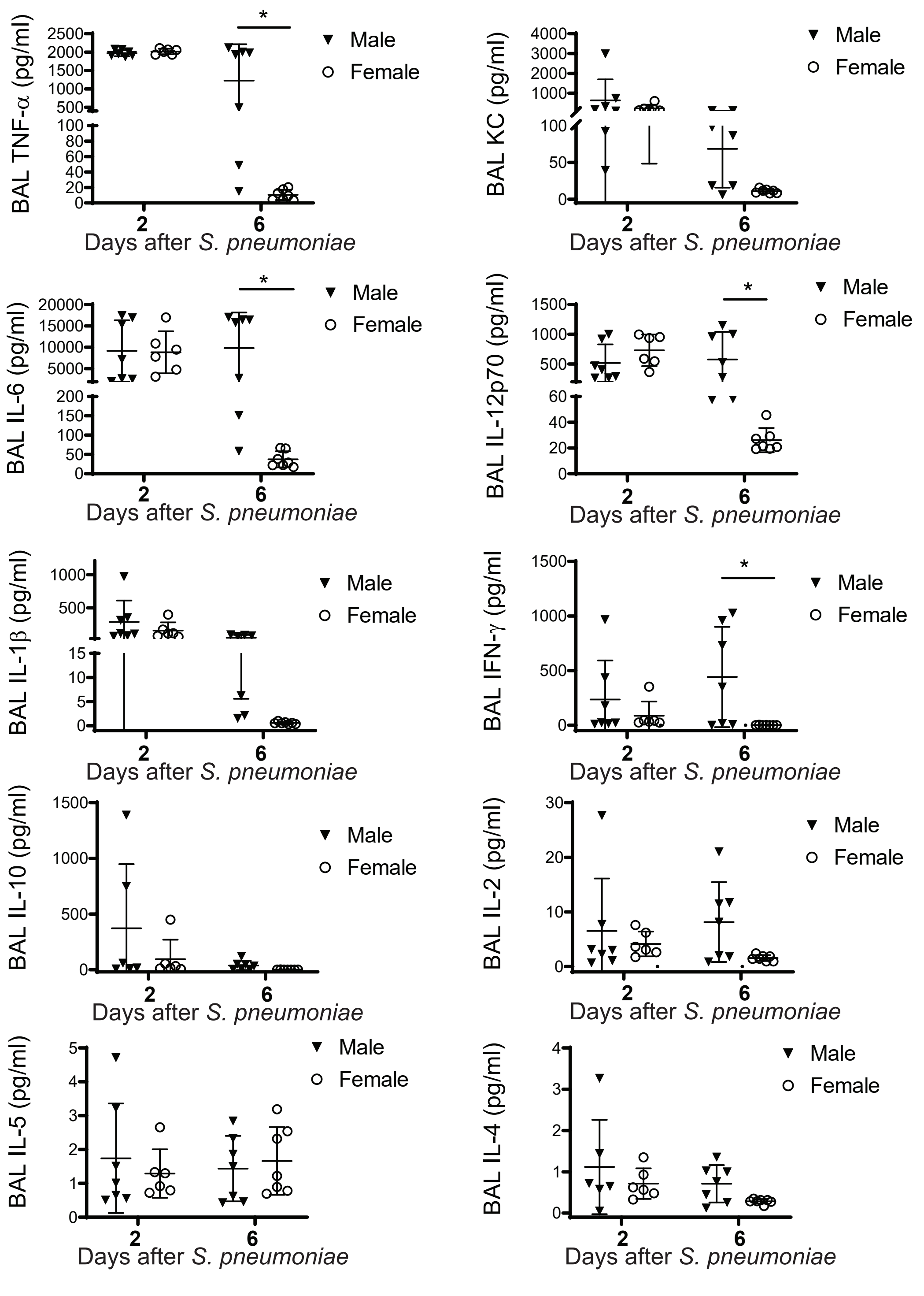

Supplementary Fig. E2.

Alveolar Inflammatory milieu in males and females after Pneumonia. Age-matched WT male and female mice were challenged with intratracheal *S. pneumoniae* (4 x 10^6 CFU/mouse) and followed over time. BAL cytokines were measured using MSD platform on days 2 and 6 after pneumonia. Two-way ANOVA was used for statistics. Mice, n=6-7 per group per time point. *P <0.05. Values reported as mean ± SEM.

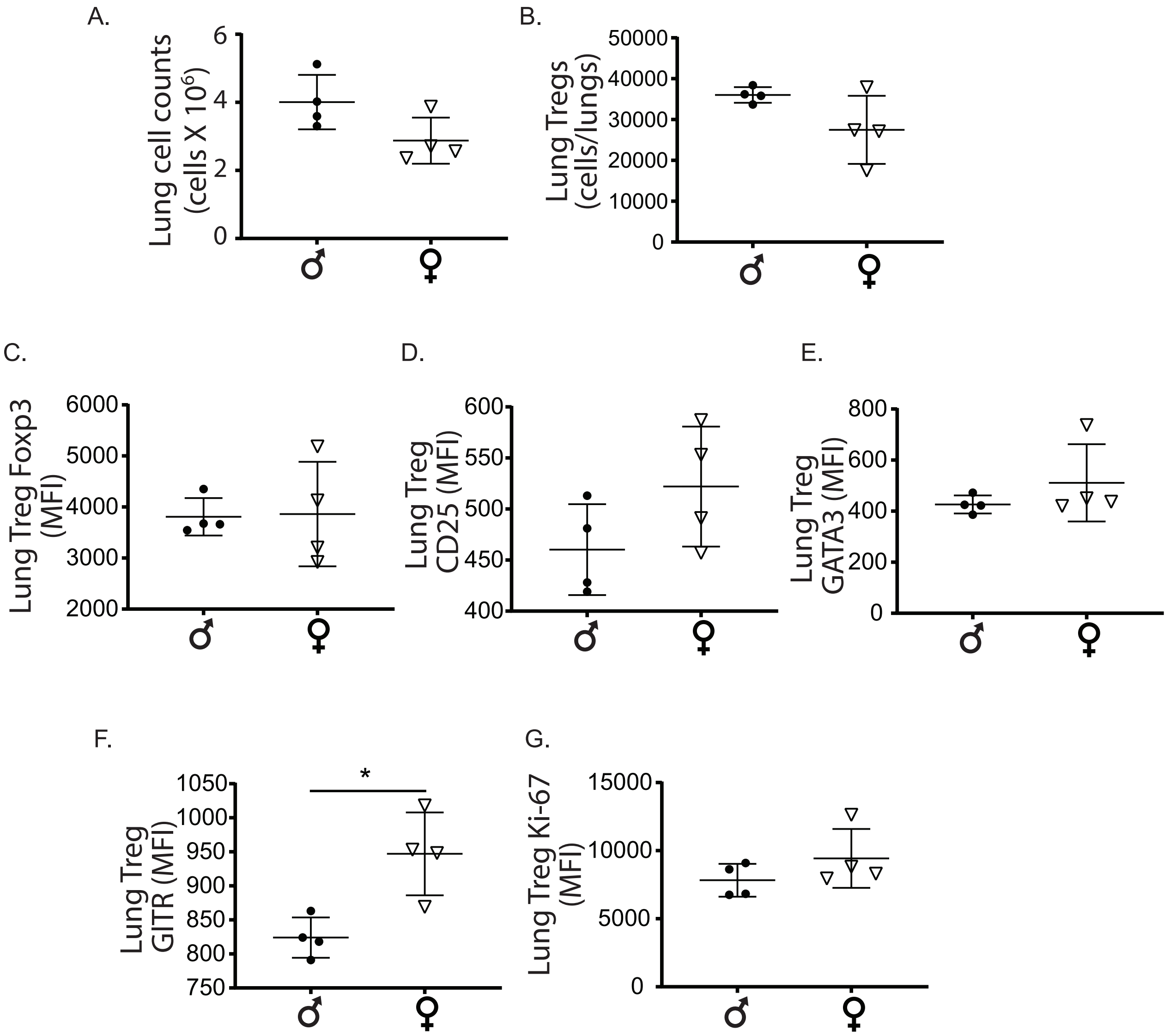

Supplementary Fig. E3

Female and male mice displayed similar lung Treg phenotype under baseline conditions. Age-matched WT male and female mice were challenged with vehicle and harvested on day 0. Lung total cell were counted in both groups (A). Multicolor flow cytometry was performed to assess lung Tregs counts and Treg suppressive phenotype. Treg expression for Foxp3 (B), CD25 (C), GATA3 (D), GITR (F) and their baseline proliferative state by Ki-67 expression (G) were measured and expressed as mean fluorescence intensity (MFI +/- SEM). Two-tailed t test was used for cell counts and the Mann-Whitney U test was used for MFI. *P <0.05.

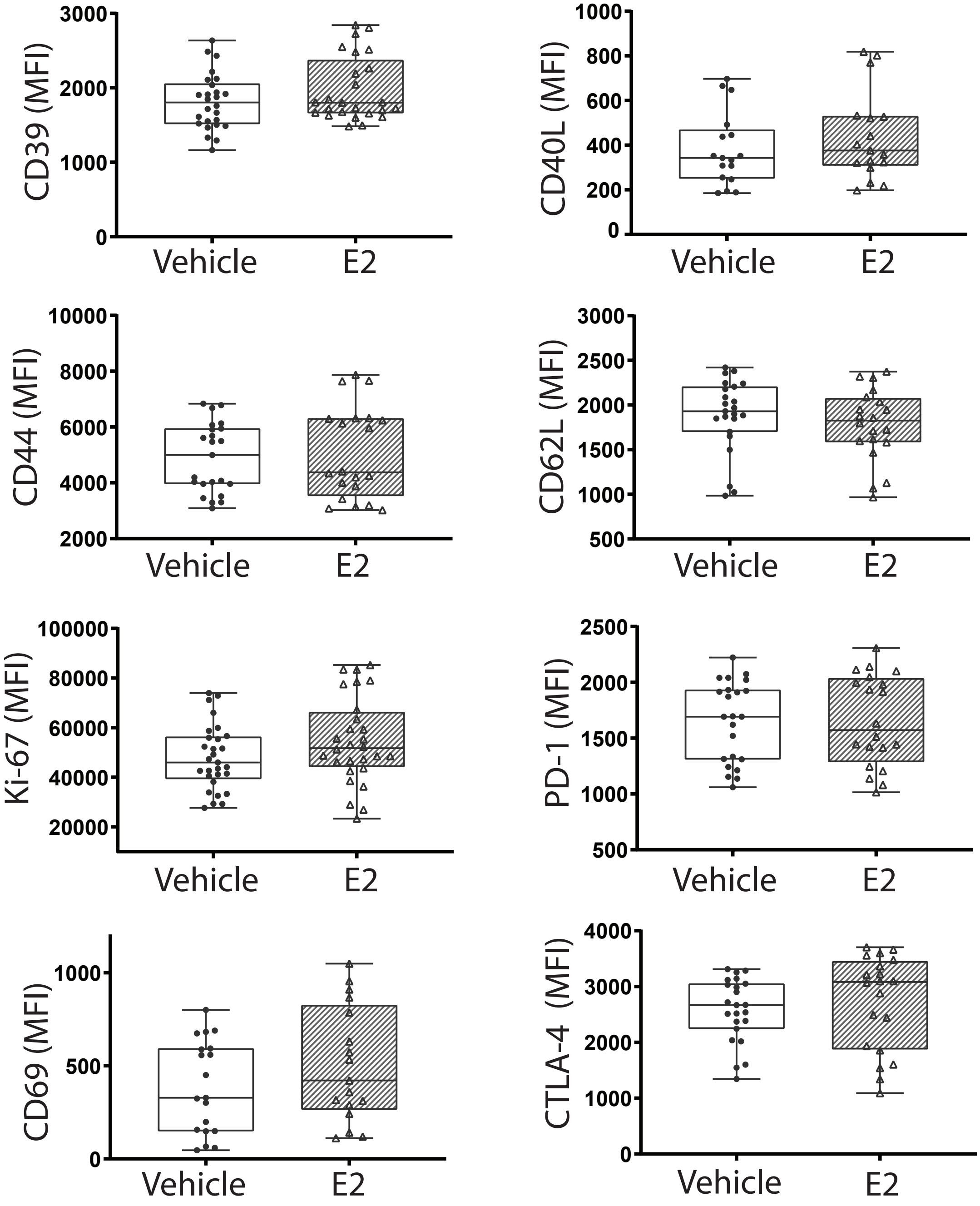

Supplementary FIG. E4

<u>Estrogen had no effects on specific Treg phenotype.</u> Male WT Tregs were isolated and cultured in the presence of anti CD3/CD28 beads and stimulated with either vehicle or estradiol (E2; 10 μM) for 72 hours. Multicolor flow cytometry was performed to asses E2 dependent changes in Treg suppressive phenotype. Treg expression for CD39, CD40L, CD44, CD62L, Ki-67, PD-1, CD69, CTLA-4 were measured and expressed as mean fluorescence intensity (MFI +/- SEM). The Mann-Whitney test was used for all MFI. *P <0.01 n =25-30 per group.

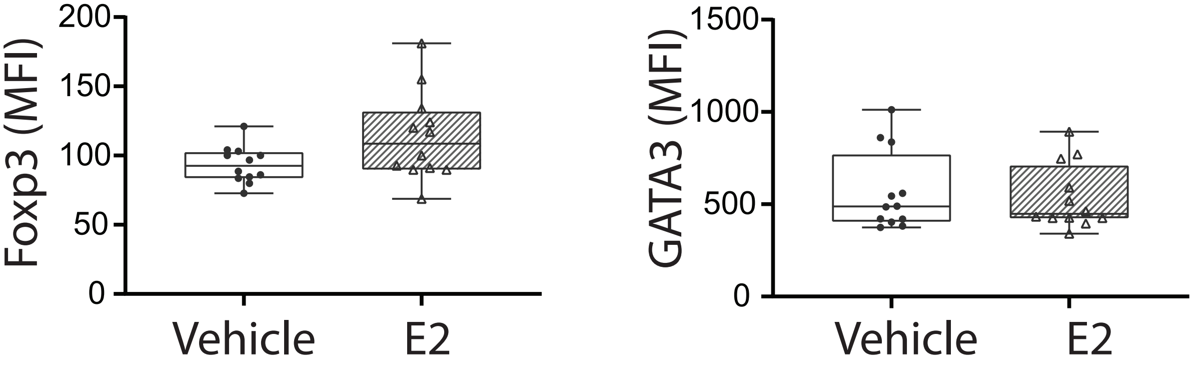

Supplementary Fig. E5.

Estrogen failed to upregulate expression of Foxp3 and GATA3 on T conventional CD4+ cells. WT CD4+CD25- were isolated and cultured in the presence of anti CD3/CD28 microbeads and stimulated with either vehicle or estradiol (E2; 10 μM) for 72 hours. Multicolor flow cytometry was performed to asses conventional for Foxp3 and GATA-3 expression were measured as mean fluorescence intensity (MFI +/- SEM). The Mann-Whitney test was used for all MFI. n = 10-12 per group.

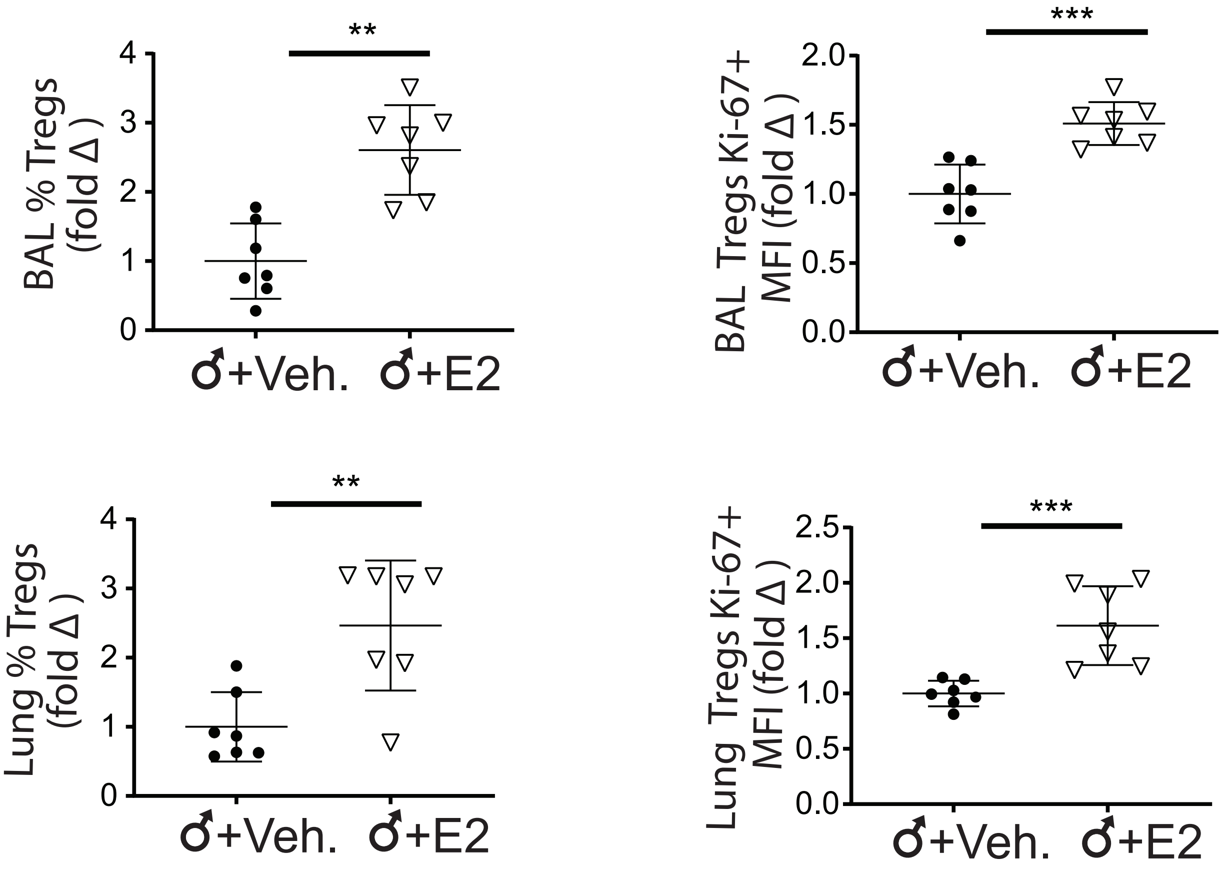

Supplementary Fig. E6

Therapeutic estradiol increased Treg ratio and proliferation in male WT animals. WT male mice were challenged with intratracheal *S. pneumoniae* (4 x 10^6 CFU/mouse). On day 2 after injury, rescue treatment with intraperitoneal estradiol 25 μg/mouse/dose) was administered daily on days 2, 3 and 4. Percentage and expression of Ki-67 for BAL and lung Tregs were measured by multicolor flow cytometry on day 6 after lung injury. Normalization to fold change followed by Mann-Whitney test was used for protein and cell counts. *P <0.05, n = 7 per group.

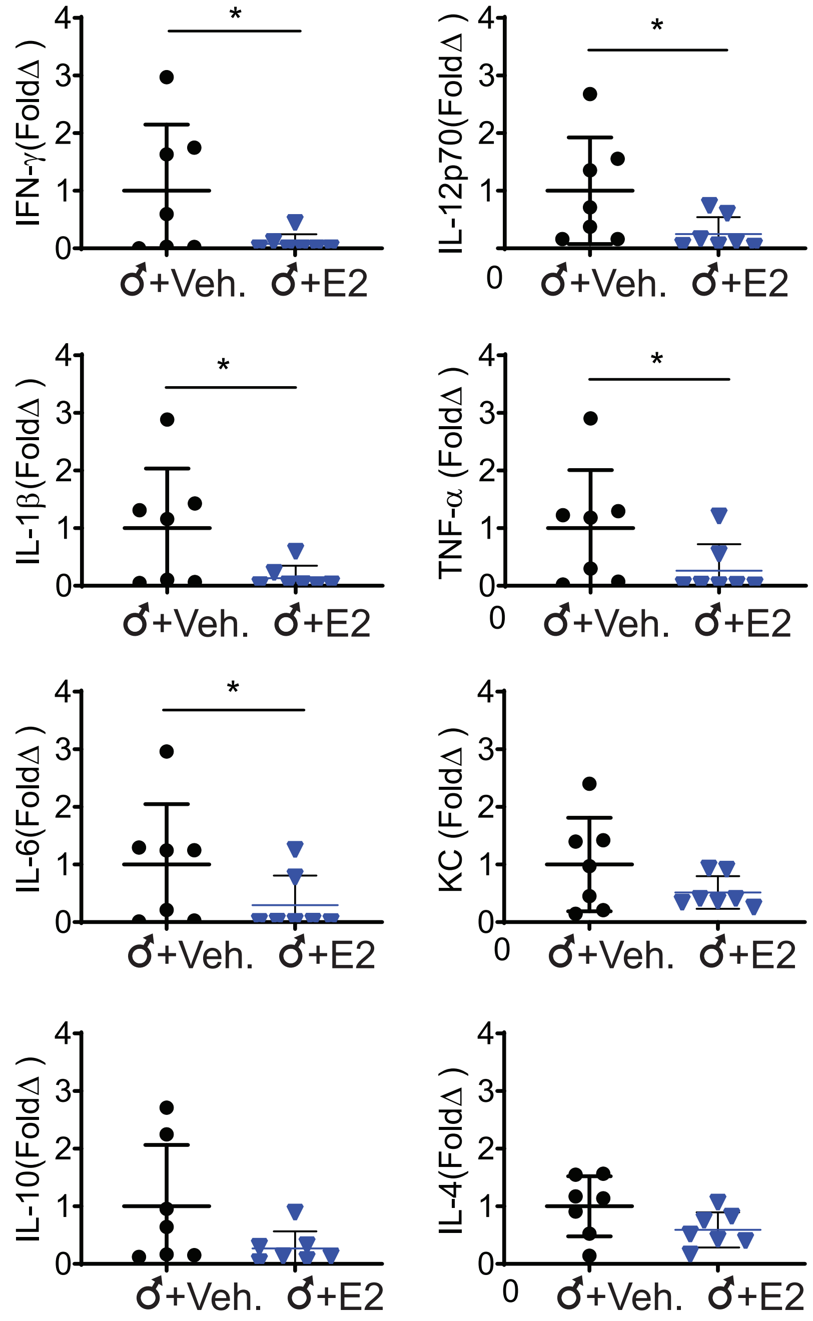

Supplementary Fig. E7.
<u>Therapeutic estradiol modulates the alveolar inflammatory milieu in males.</u> WT male mice were challenged with intratracheal S. pneumoniae (4 x 10^6 CFU/mouse). On day 2 after injury, rescue treatment with intraperitoneal estradiol (25 μg/mouse/dose) was administer daily on days 2, 3 and 4. BAL cytokines were measured using MSD platformon day 6 after lung injury. Normalization to fold change followed by Mann-Whitney test was used for protein and cell counts. *P <0.05, n = 7 per group.

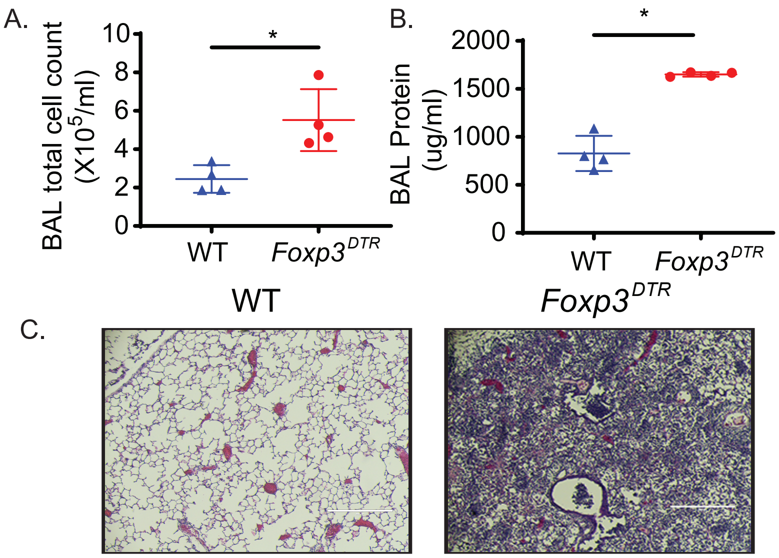

Supplementary Fig. E8.

Tregs play a critical role in resolution of *S. pneumoniae*-induced pneumonia. Male WT and *Foxp3*^{DTR} mice were challenged with intratracheal *S. pneumoniae* (3 x 10^6 CFU/mouse). Both groups received diphtheria toxin (day-2 at 50 μg/kg, subsequent doses at 10 μg/kg). BAL total cell count (A) and BAL total protein (B) were determined on day 6 after intratracheal *S. pneumoniae*. Representative lung H-E section (C) shown (Magnification 40X). Paired T-test was used. Mice, n=4 per group per time point. *P <0.05. Values reported as mean ± SEM.

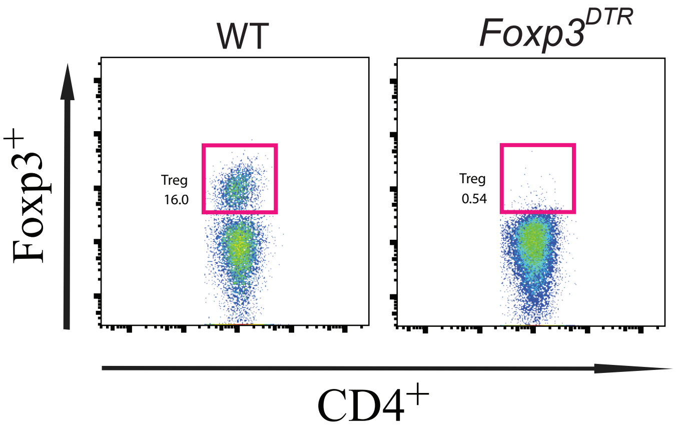

Supplemetary Fig. E9

Diphtheria toxin (DT)-treatment depleted Tregs in the lung of *Foxp3^DTR* mice but did not influence that of WT mice. Male WT and *Foxp3^DTR* mice were challenged with intratracheal *S. pneumoniae* (3 x 10^6 CFU/mouse). Both groups received DT (day-2 at 50 μg/kg, subsequent doses at 10 μg/kg). Representative flow cytometry for lung Tregs were measured by multicolor flow cytometry on day 5 after lung injury.

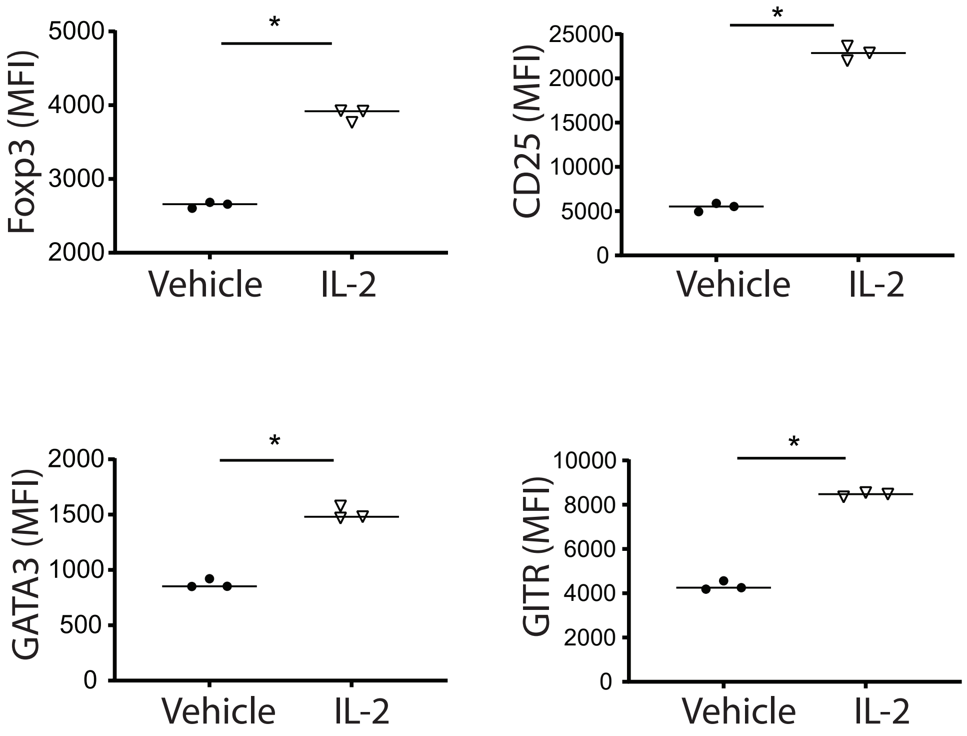

Supplementary Fig. E10

IL-2 augmented the suppressive phenotype of ER$\beta^{-/-}$ Tregs. CD4+CD25+ Tregs were isolated from males *ER*$\beta^{-/-}$ splenocytes and cultured in the presence of anti CD3/CD28 beads and stimulated with either vehicle or IL-2 (100 U/ml) for 72 hours. Multicolor flow cytometry was performed for the expression of Foxp3(A), CD25(B), GATA-3(C) and GITR(D) and measured by mean fluorescence intensity (MFI) The Mann-Whitney U test was used for all MFI. *P <0.05, (n = 3 per group).

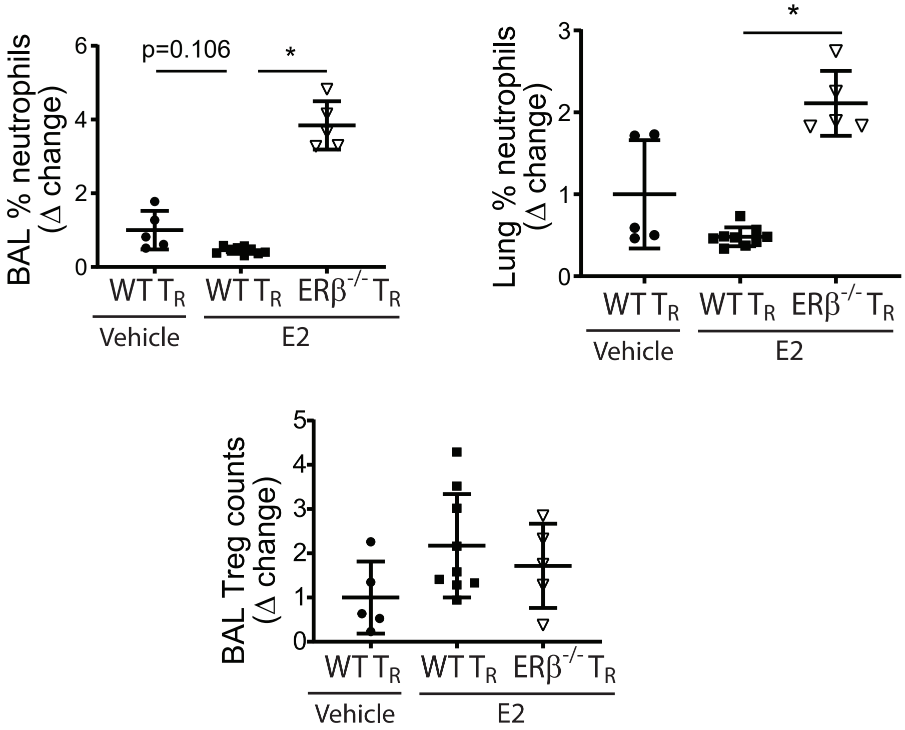

Suppplementary Fig. E11.

<u>Estradiol augmented Treg function in an ERβ-dependent manner.</u> Male WT and *ER*β$^{-/-}$ Tregs were cultured in the presence of anti CD3/CD28 beads and stimulated with either vehicle or estradiol (E2; 10 μM) for 48 hours. Cells were collected and 0.25 X 10^6 Tregs adoptively transferred (AT) (retro-orbital) 1 hour after intratracheal *S. pneumoniae* (3 X 10^6 CFU/mouse) in lymphocyte-deficient *Rag-1*$^{-/-}$ mice. BAL and lung neutrophil percentage as well as BAL Treg counts were measured at day 5 and expressed as fold change compared to group of *Rag-1*$^{-/-}$ mice AT with WT Tregs cultured *ex vivo* with vehicle (ethanol). Normalization followed by Kruskal-Wallis test was used for statistics. *P <0.05, values reported are mean ± SEM.

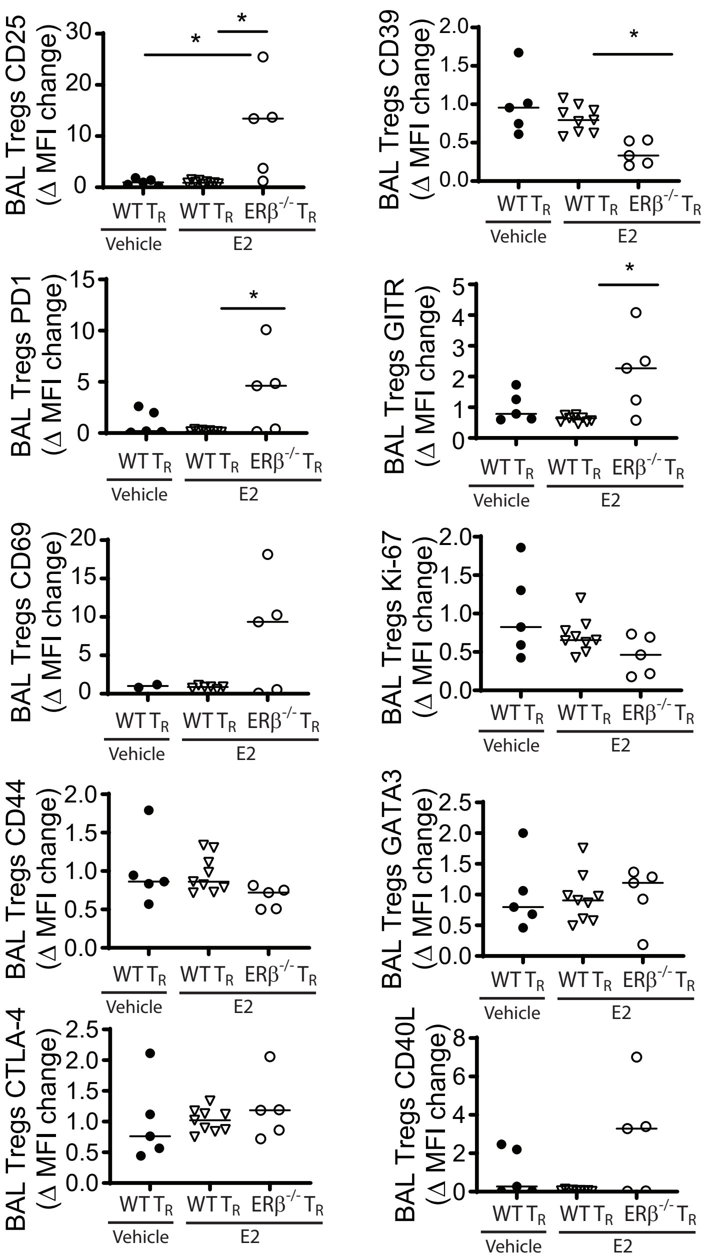

SUPPLEMENTARY FIG. E12.

Male WT and *ER*β-/- Tregs were cultured in the presence of anti CD3/CD28 beads and stimulated with either vehicle or estradiol (E2; 10 μM) for 48 hours. Cells were collected and 0.25 X 10^6 Tregs adoptively transferred (AT) (retro-orbital) 1 hour after intratracheal *S. pneumoniae* (3 X 10^6 CFU/mouse) in lymphocyte-deficient *Rag-1*$^{-/-}$ mice. BAL Treg phenotype were measured at day 5 and expressed as fold change compared to group of *Rag-1*$^{-/-}$ mice AT with WT Tregs cultured *ex vivo* with vehicle (ethanol). Normalization followed by Kruskal-Wallis test was used for statistics. *P <0.05, values reported are mean ± SEM.

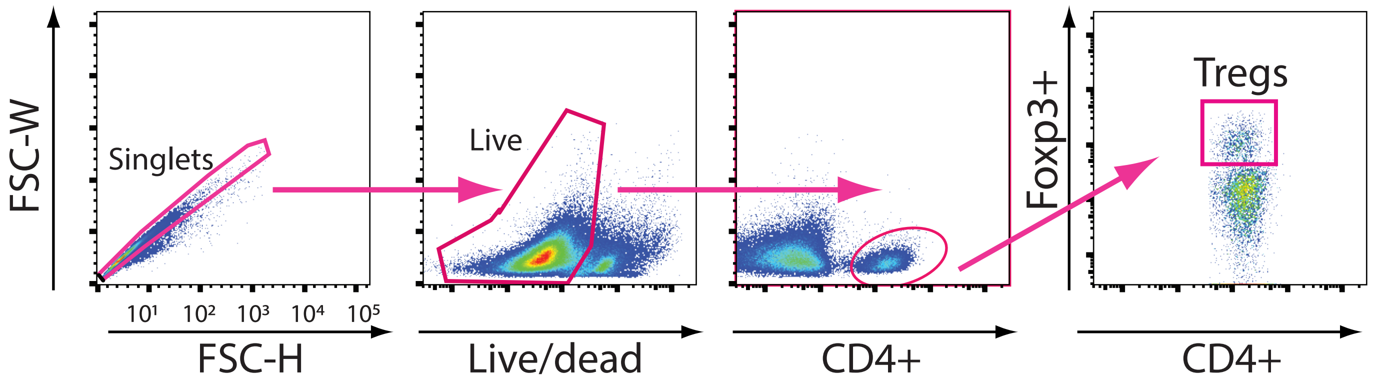

Supplementary Fig. E13
Flow cytometry gating strategy. Singlets and doublets were excluded. Subsequently dead cell were gated out followed by CD4+ gating. CD4+Foxp3+ were used to identify Regulatory T cells (Tregs). Tregs were gated for the relative expression of suppressive markers using fluorescence minus one (FMO; not shown).
